# Inhibition of Defect-Induced Ice Nucleation, Propagation, and Adhesion by Bioinspired Self-Healing Anti-Icing Coatings

**DOI:** 10.34133/research.0140

**Published:** 2023-05-18

**Authors:** Shu Tian, Ruiqi Li, Xinmeng Liu, Jiancheng Wang, Junyu Yu, Sijia Xu, Yunqing Tian, Jing Yang, Lei Zhang

**Affiliations:** ^1^ Department of Biochemical Engineering, Frontier Science Center for Synthetic Biology and Key Laboratory of Systems Bioengineering (MOE), School of Chemical Engineering and Technology, Tianjin University, Tianjin 300350, China.; ^2^ Binzhou Institute of Technology, Weiqiao-UCAS Science and Technology Park, Binzhou City, Shandong Province 256606, China.

## Abstract

Anti-icing coatings on outdoor infrastructures inevitably suffer from mechanical injuries in numerous icing scenarios such as hailstorms, sandstorms, impacts of foreign objects, and icing–deicing cycles. Herein, the mechanisms of surface-defect-induced icing are clarified. At the defects, water molecules exhibit stronger adsorption and the heat transfer rate increases, accelerating the condensation of water vapor as well as ice nucleation and propagation. Moreover, the ice–defect interlocking structure increases the ice adhesion strength. Thus, a self-healing (at −20 °C) antifreeze-protein (AFP)-inspired anti-icing coating is developed. The coating is based on a design that mimics the ice-binding and non-ice-binding sites in AFPs. It enables the coating to markedly inhibit ice nucleation (nucleation temperature < −29.4 °C), prevent ice propagation (propagation rate < 0.00048 cm^2^/s), and reduce ice adhesion on the surface (adhesion strength < 38.9 kPa). More importantly, the coating can also autonomously self-heal at −20 °C, as a result of multiple dynamic bonds in its structure, to inhibit defect-induced icing processes. The healed coating sustains high anti-icing and deicing performance even under various extreme conditions. This work reveals the in-depth mechanism of defect-induced ice formation as well as adhesion, and proposes a self-healing anti-icing coating for outdoor infrastructures.

## Introduction

Throughout the story of human survival, the selection or design of well-suited materials inspired from nature has been important to withstand environmental challenges [1–5]. For example, to cope with humid situations, duck feathers were used to resist water penetration at the initial stages of exposure to humidity, whereas lotus leaf-inspired superhydrophobic surfaces (SHSs) were developed to completely repel water in recent years [6–9]. Currently, anti-icing coatings based on the principle of water repellency have been developed to resist the detrimental effects of ice accumulation on surfaces. The complex surface structure of SHSs enhances their repellency performance, while bringing the problems caused by the voids on the surface under frost conditions [10–12]. Slippery liquid-infused porous surfaces (SLIPSs) are a new class of *Nepenthes* pitcher-inspired materials based on textured solids infiltrated with lubricants. SLIPSs were created to address the void problem and expand the potential application of surface fabrication technologies [13–16]. The recently advanced slippery surfaces exhibit multifunctional anti-icing performance for potential usage [17,18].

However, anti-icing coatings for large-scale outdoor infrastructures still face substantial challenges [19,20]. First, water repellency alone is not sufficient for anti-icing coatings used in several open-air icing scenarios. The coatings are inevitably subjected to harsh situations, such as hailstorms, sandstorms, foreign-object impacts, and icing–deicing cycles, resulting in mechanical injuries to their surfaces [21–23]. Second, the textured surfaces are hard to fabricate for large-scale infrastructures. Therefore, effective open-air anti-icing coatings should suppress ice formation, reduce ice adhesion, and preferably self-heal (in a similar fashion to mammalian skins) to resist mechanical injuries [24–26]. Moreover, outdoor operability and stability of the coatings must be considered [9,15,27].

**Fig. 1.**
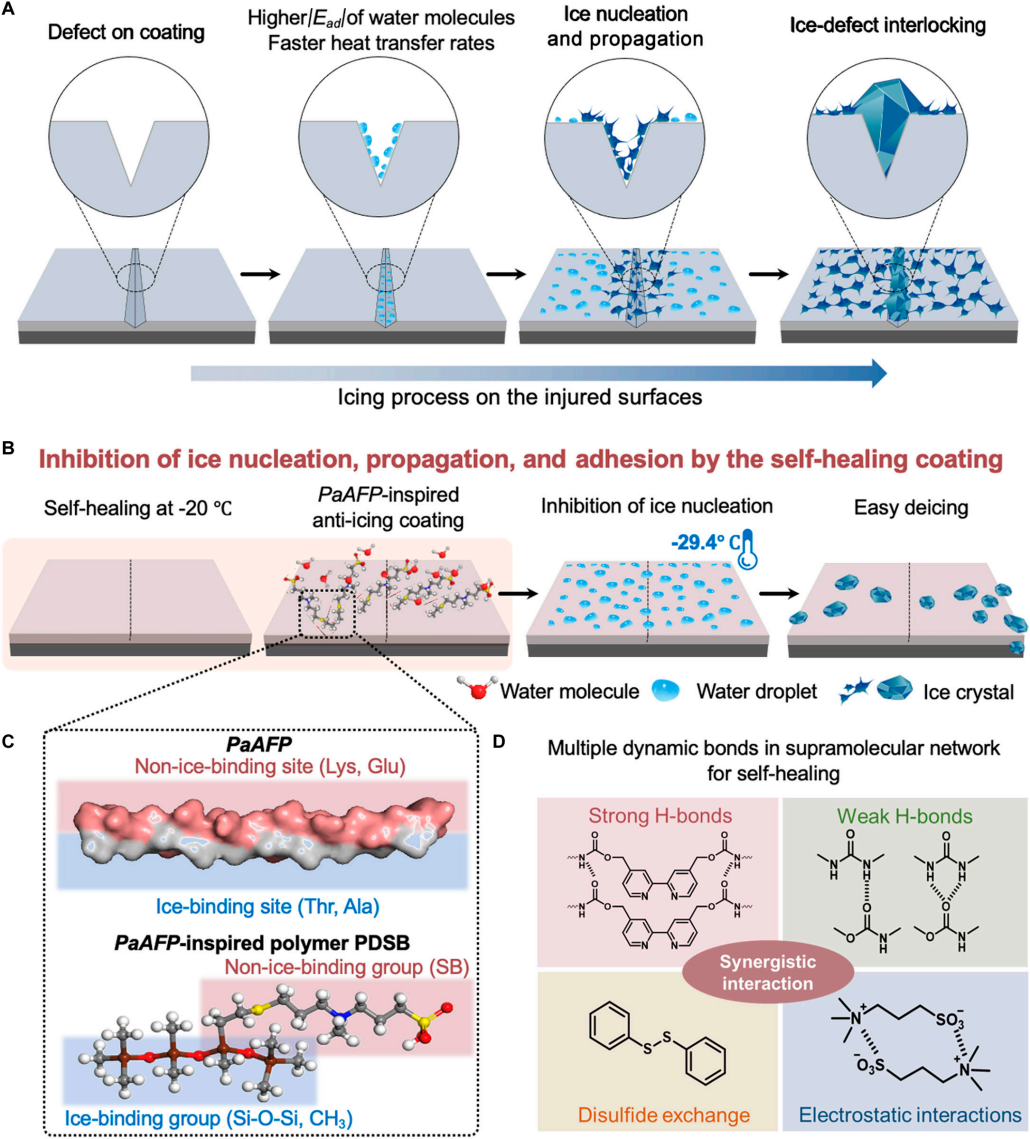
Schematic illustrations of the (A) icing processes on the injured surfaces, and (B) ice inhibition by the bioinspired self-healing anti-icing coating at −20 °C. (C) Design of the PDSB polymer, which contains IBS and NIBS to mimic the *Pseudopleuronectes americanus* antifreeze-protein (*Pa*AFP). (D) Synergistic interactions of multiple dynamic bonds in the supramolecular network of the self-healing coating.

Natural antifreeze proteins (AFPs) help polar fish avoid freezing [28]. Herein, a self-healing AFP-inspired coating with both high anti-icing and deicing abilities was developed. These abilities completely originate from the intrinsic properties of the materials instead of the surface microstructure, of which the anti-icing properties are mainly based on water repellency. First, the mechanisms of the defect-induced icing on the coating surfaces, which were rarely studied, were clarified, revealing the importance of self-healing anti-icing coatings (Fig. 1A). Next, based on the understanding of key antifreeze characteristics in AFPs, a poly(dimethylsiloxane-co-sulfobetaine methacrylate) (PDSB) copolymer was designed to mimic their non-ice-binding sites (NIBS) and ice-binding sites (IBS), providing the coating with the ability to inhibit ice nucleation, prevent ice propagation, and reduce ice adhesion strength (Fig. 1B and C) [29,30]. Moreover, the PDSB-based coating was designed with remarkable self-healing properties even at −20 °C to inhibit defect-induced icing processes. This ability can be attributed to the synergistic interaction of multiple dynamic bonds in a supramolecular network matrix (Fig. 1D). This work provides a basis for the large-scale fabrication of self-healing anti-icing coatings suitable for complex environments.

## Results

### Defect-induced icing processes

Outdoor anti-icing coatings are inevitably subjected to mechanical injuries caused by storm, foreign-object impacts, and icing–deicing cycles. Defects on the coating surfaces reduce their anti-icing performance and even promote the icing process. Here, the defect-induced icing promotion mechanism was clarified to reveal the importance of self-healing coatings in practical applications.

**Fig. 2.**
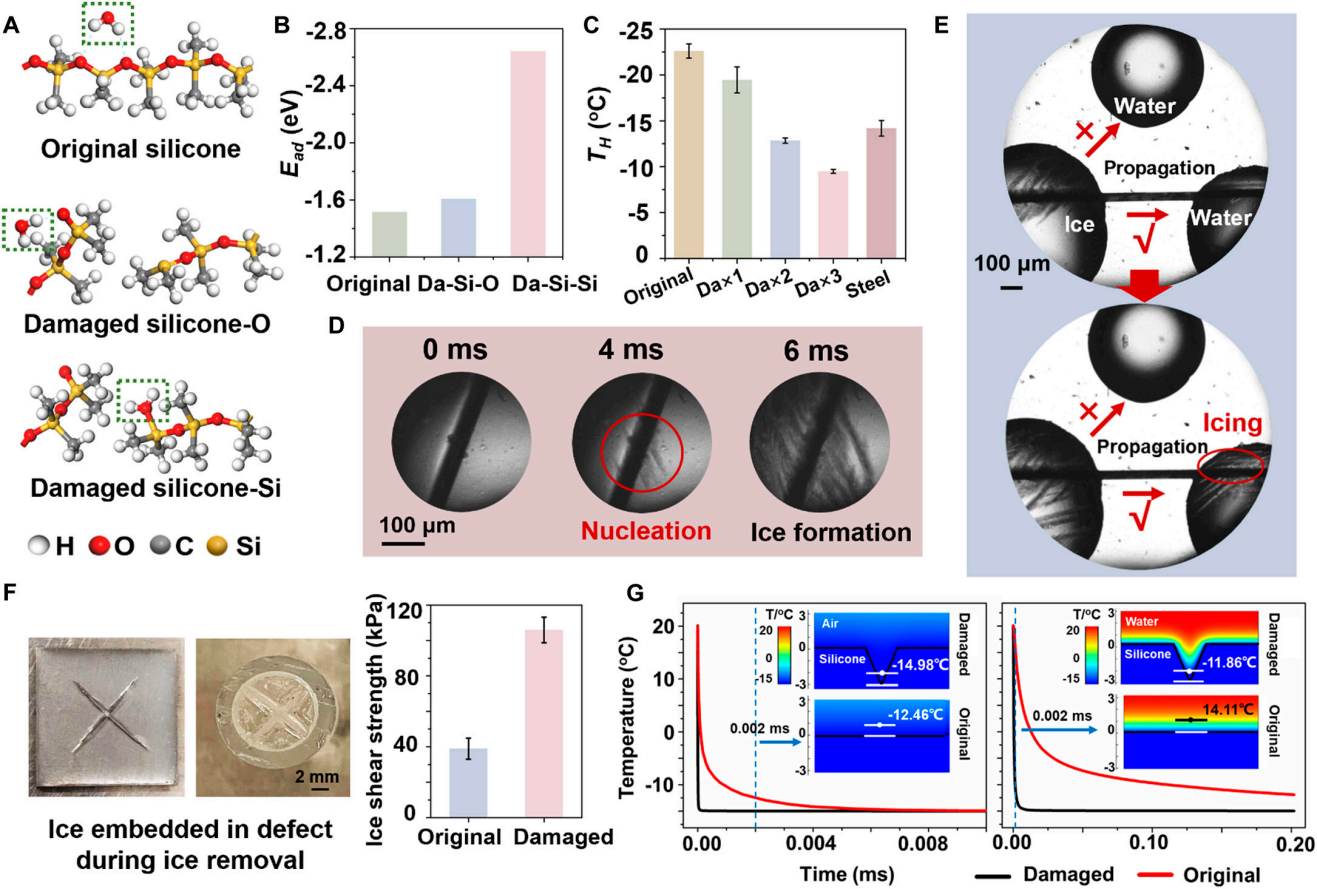
Defect-induced ice nucleation, propagation, and adhesion. (A) The DFT-optimized structures and (B) *E_ad_* of a single H_2_O molecule on intact and damaged silicone chains. (C) *T_H_* of an intact PDMS coating and of that with 1, 2, and 3 defects. Da, Damaged. (D) Optical microscopic images of ice nucleation on the coatings with defect. (E) Optical microscopic images of ice propagation from a frozen droplet to another droplet via a defect acting as an ice bridge. (F) The images of the damaged coating and defect structure on the bottom of an icicle stripped from the coating (left), and the ice shear strength of the coating before and after damage (right). (G) Time–temperature curves of the coating–air system (left) and coating–water system (right).

An unhealable pristine polydimethylsiloxane (PDMS) matrix, similar to the developed self-healing anti-icing PDMS-based coating matrix, was used to investigate the defected coating model in the following tests. First, the adsorption energy (*E_ad_*) of a water molecule at a defect was calculated using density functional theory (DFT) simulations. The PDMS coating was represented by a single silicone chain, whereas defects were modeled using a broken silicone chain containing a broken Si–Si or Si–O bond (Fig. 2A). As shown in Fig. 2B, the absolute value of *E_ad_* of an H_2_O molecule on a silicone chain was 1.51 eV, whereas those on O and Si atoms in a broken Si–O–Si bond were higher (1.61 and 2.64 eV, respectively). This indicates that the water adsorption on the surface defect is markedly promoted, leading to preferential ice crystallization [31,32].

Experimental icing tests of the original PDMS coatings and those with surface defects, including water condensation, ice nucleation, propagation, and adhesion, were systemically performed. The coatings were placed in a closed cell (humidity = 50%) and cooled from 20 °C to −15 °C before observed. During the cooling process, water was preferentially condensed at the defect, at which it was susceptible to freezing (Fig. S1). The heterogeneous ice nucleation (HIN) temperatures (*T_H_*) of the water droplets on the coatings were investigated. According to previous reports, when the cooling rate is 5 °C/min, the *T_H_* of water could be considered as the temperature of the whole water droplet that was frozen within 1 s. As shown in Fig. 2C, Fig. S2, and Movie S1, the *T_H_* of the water droplet on the intact PDMS coating surface was −22.6 °C, whereas that on the coating with 1, 2, and 3 defects were −19.5 °C, −12.9 °C, and −9.5 °C, respectively. The *T_H_* on the coating with 3 defects was even higher than that on an uncoated steel surface (−13.6 °C), confirming the defect-induced icing phenomenon. The ice nucleation process was also observed on the surface with defects using a high-speed camera (Fig. 2D and Movie S2). The initial ice nucleation occurred at the defect, and ice diffused and formed until the whole droplet was completely frozen. The icing delay of the water droplets on the coating surfaces illustrated in Fig. S3 indicates that water started to freeze on the intact surface after 55 s, whereas after only 3 s did water freeze on the surfaces with defects. These results confirm that the defect-induced ice nucleation could markedly increase *T_H_* and promote the freezing of water droplets.

Ice propagation was then investigated on the intact surfaces and those with defects. Ice from a frozen droplet propagate to other droplets through ice bridges [33]. As shown in Fig. 2E and Movie S3, the frozen droplet preferentially propagated to induce freezing of another droplet through the defect that provided a bridge for ice propagation. Subsequently, a nucleating agent (AgI nanoparticles) was used on the coating surface with defects, and the ice propagation process of a water droplet on the surface was observed. As shown in Fig. S4 and Movie S4, tiny ice crystals occurred at the defect and further propagated to the whole droplet along the defect. Moreover, defect-induced ice adhesion on the coating surfaces was evaluated. Figure 2F shows the defect structure on the bottom of an icicle stripped from the coating, and the ice shear force on the coating with defects markedly increased from 38.9 to 105.9 kPa. This indicates that the ice–defect interlocking structure increases the ice adhesion strength and reduces the deicing performance of the coating.

Next, an in-depth investigation of the defect-induced icing mechanism was conducted by simulating the heat transfer process on the intact coatings and those with defects using finite element simulations. Figure 2G shows the heat transfer process (from 20 °C to −15 °C) at the coating–air and coating–water interfaces. After 0.002 ms of cooling, the air temperature at 1 μm above the defect decreased to −14.98 °C, whereas that above the intact coating was −12.46 °C. The results indicated that the cooling rate of the air above the damaged surface was markedly higher, leading to the preferential condensation of the water vapor at the defects, consistent with the above-mentioned experimental results. At the coating–water interface, the cooling rate of water on the defects was much higher than that on the intact coating, indicating the preferential crystallization of water on the coating with defects. In addition, the heat transfer rate was directly proportional to the defect depth (Fig. S5).

Overall, the defect-inducing icing mechanisms were systemically investigated and clarified. At the defects, higher heat transfer rates and absolute value of *E_ad_* of the H_2_O molecule cause a preferential condensation of the water vapor for ice nucleation and formation. Small ice crystals can propagate via the defect, which acts as an ice bridge, finally leading to a massive ice accretion on the surface with defects. In addition, the ice–defect interlocking structure induces very high ice adhesion strength, which hiders deicing performance. Moreover, the defect-induced icing was further verified on other surfaces, including steel, glass, and epoxy resin, as shown in Fig. S6.

### Design of AFP-mimic PDSB

**Fig. 3.**
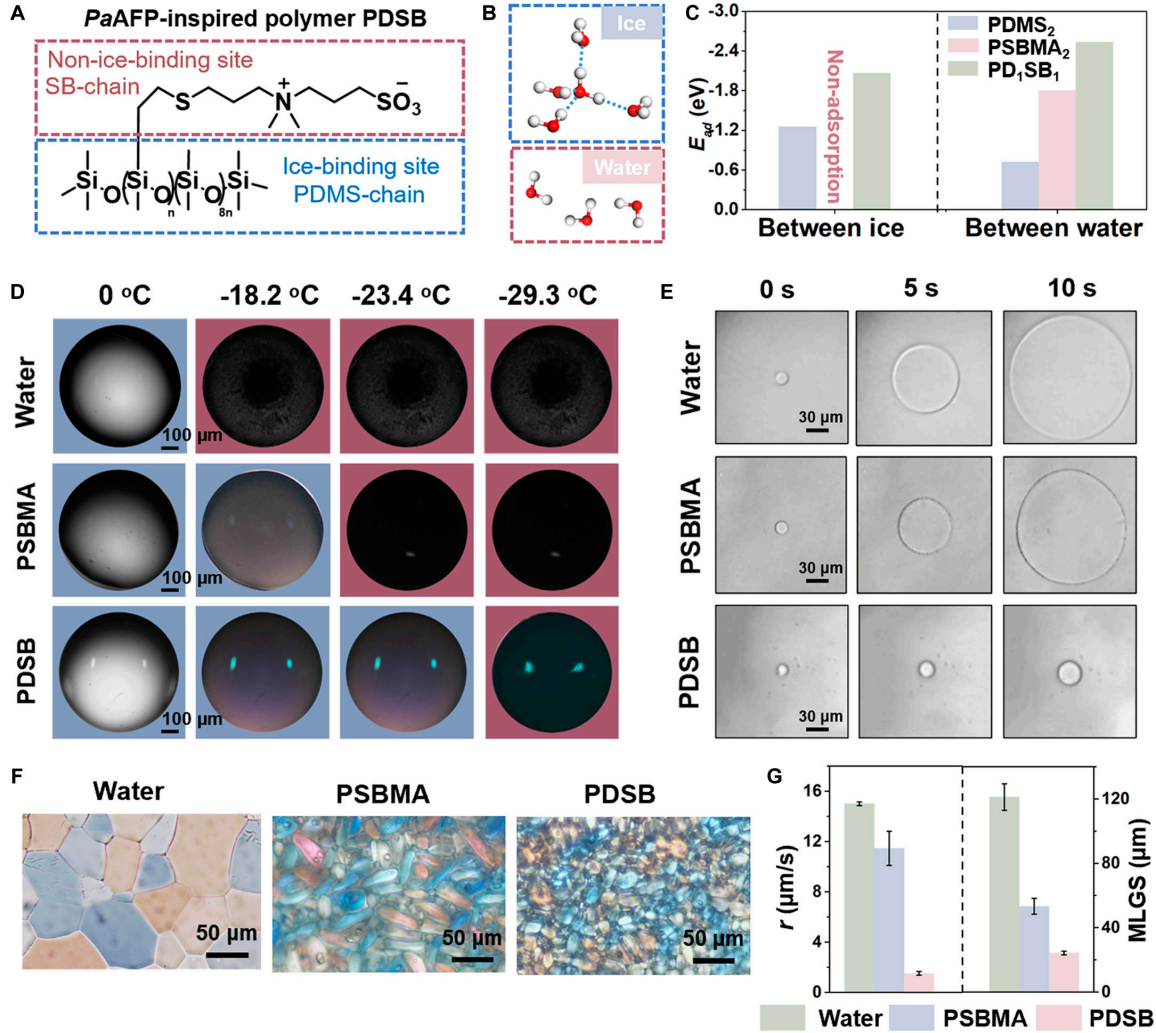
Design and antifreeze properties of AFP-mimic PDSB. (A) *Pa*AFP-mimic NBS and IBS structure of PDSB. (B) Hexagonal ice model (ice Ih) and water molecules used for the calculation of (C) *E_ad_* on PDMS_2_, PSBMA_2_, and PD_1_SB_1_. Ice Ih: each O atom is surrounded by 4 O atoms in a tetrahedral coordination, and H atoms are distributed in an orderly manner. (D) Optical microscopic images of pure water, PSBMA solution (100 mg/ml), and PDSB solution (100 mg/ml) droplets freezing at different temperatures. (E) Morphology of a single ice crystal growth at different periods in pure water, PSBMA, and PDSB solution. (F) Optical microscopic images of the recrystallized ice crystals in pure water, PSBMA solution, and PDSB solution. (G) Growth rates of a single ice crystal and the MLGSs of recrystallized ice crystals in water, PSBMA solution, and PDSB solution.

Natural AFPs can effectively control ice formation as a result of their Janus effect [34,35]. The IBS of AFPs can adsorb/bind to ice crystals to bend its surface and inhibit its growth due to the Kelvin effect. Moreover, the NIBS of AFPs can form a hydration layer with a disordered structure to depress ice nucleation. The IBS of *Pa*AFP contain specific spatial arrangement of hydroxyl and methyl groups, whereas NIBS consist of charged groups [29,30]. Herein, a PDSB copolymer with IBS-mimic PDMS and NIBS-mimic poly(sulfobetaine methacrylate) (PSBMA) segments was developed (Fig. 3A and Fig. S7). Figure S8 shows the synthetic route and ^1^H nuclear magnetic resonance (NMR) spectrum characterization of PD*_n_*SB*_m_*, and Fig. S9 shows the Fourier-transform infrared spectrum and X-ray photoelectron spectroscopy (XPS) results of PDSB.

To verify the AFP-mimic design of PDSB, DFT simulation was performed to investigate the *E_ad_* of ice/water molecules on PDMS, PSBMA, and PDSB. In addition, the copolymers were simplified to PDMS_2_, PSBMA_2_, and PD_1_SB_1_ shown in Fig. S10 for DFT simulation. The most ordinary hexagonal ice model (ice Ih) was used in this simulation (Fig. 3B) [36]. As shown in Fig. 3C, the absolute value of *E_ad_* of ice and water molecules on a PDMS_2_ chain was 1.25 and 0.72 eV, respectively, indicating the higher energy stability of ice crystals on PDMS_2_ than that of water on it. An IBS-mimic mechanism, in which the spacing between the O atoms in the Si–O–Si chain (2.659 Å) well matched the crystal lattice spacing of ice (2.698 Å), was proposed (Fig. S11A) [37,38]. The hydrophobic methyl group could regulate the amount of interfacial water, causing an effective binding of ice crystals [5]. Therefore, the ice crystals in PDSB solutions showed an apparent hexagonal shape under Δ*T* = 0.06 °C (Fig. S11b). The absolute value of *E_ad_* of the water molecules on PSBMA_2_ (Fig. 3C) was 1.80 eV, while ice crystal was completely not adsorbed on PSBMA_2_, confirming that it has NIBS-mimic properties. Zwitterionic sulfobetaine (SB) segments can bind to water molecules through their ionic solvation effects and H bonds in molecular dynamics (MD) simulation results, thus reducing the water freezing point and inhibiting ice nucleation (Fig. S12 and Movie S5) [39,40]. Moreover, the absolute value of *E_ad_* of ice and water molecules on PD_1_SB_1_ was 2.06 and 2.53 eV, respectively, suggesting its strong binding force with both ice crystals and water molecules compared to the binding forces of PDMS_2_ with ice and PSBMA_2_ with water molecules, respectively. Therefore, PDSB can effectually mimic the IBS and NIBS of AFPs to achieve good antifreeze performance.

Next, the antifreeze properties of PDSB, i.e., its ability to inhibit ice nucleation, crystal growth, and recrystallization, were systemically investigated. As shown in Fig. 3D, the *T_H_* of a PDSB solution (100 mg/ml) was −29.4 °C, which is much lower than that of a 100-mg/ml PSBMA solution (−23.4 °C) and pure water (−18.2 °C), demonstrating the ability of AFP mimetics to inhibit ice nucleation. Figure 3E and G show that the growth rates of a single ice crystal during the freezing process decreased from 15.0 to 1.5 μm/s after the addition of PDSB, which is highly superior to that obtained by the PSBMA addition (11.5 μm/s). The results indicate that PDSB could substantially interact with the crystal surface to inhibit its growth. During the thawing process, Fig. 3F and G show that the mean largest grain size (MLGS) of the recrystallized crystals of pure water after annealed 30 min was 121.18 μm, whereas the MLGSs of PSBMA and PDSB samples were 53.25 and 24.23 μm, respectively. The above results indicated that PDSB could effectively inhibit ice nucleation, crystal growth, and recrystallization. These abilities of PDSB can be attributed to its unique structure with IBS and NIBS similar to those of AFPs. The inhibition of ice nucleation on a surface would prevent the supercooled water from freezing, effectively delaying icing, whereas the inhibition of ice crystal growth and recrystallization would effectively prevent the rapid ice propagation and aggregation on the surface.

### Anti-icing/deicing performance of the AFP-inspired coating

PDSB with an optimal PDMS/PSB ratio was added into a silicone-based supramolecular polymeric (PSIB) matrix to develop a self-healing anti-icing coating. Four ratios of PSIB-PDSB coatings were prepared and the proportions of each coating are shown in Table S1. Figure S14 shows the XPS spectra of the developed anti-icing coatings, illustrating the efficient integration of PDSB into the PSIB matrix. The morphologies and structure of the anti-icing coatings were further studied by scanning electron microscopy, mapping, differential scanning calorimetry, and tensile tests (Figs. S15 and S16). These tests indicated that the coatings were uniform with supramolecular polymer networks and without apparent phase separation [41,42]. In addition, the anti-icing coatings presented the high adhesion level (5B) on various substrates, including steel, glass, and epoxy primer, according to ASTM D3359 (Fig. S17).

**Fig. 4.**
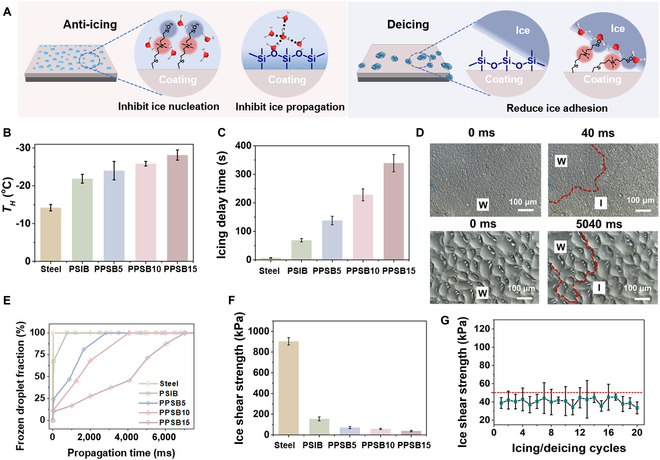
Anti-icing/deicing performance of the AFP-inspired coating. (A) Anti-icing and deicing mechanisms of the PPSB coating. (B) *T_H_* and (C) icing delay time on the uncoated steel and on different coatings. (D) Time-resolved optical microscopic images of ice propagation on the PSIB (upper images) and PPSB15 coatings (lower images). (E) Change in the fraction of frozen droplets on different coatings. (F) Ice adhesion strength of different coatings. (G) Variation in ice adhesion strength on the PPSB15 coating over 20 icing–deicing cycles.

As shown in Fig. 4A and Fig. S18, the NIBS-mimic SB segments can migrate to the coating surface and enhance its hydrophilicity to regulate interfacial water molecules. The IBS-mimic segments (Si–O–Si) inhibited the ice propagation along the coating surface. According to the Kendall equation, the low elastic modulus (<0.3 MPa) and surface energy (<20 mJ·m^−2^) of the Si-based matrix provided the coating with a low ice adhesion strength (Fig. S19 and Supporting information note 1) [43]. Moreover, the hydrophilic SB segments generated a certain amount of non-freezable water at the ice–coating interface to further reduce the ice adhesion strength [44].

Next, the anti-icing and deicing performance of the AFP-inspired coatings were systemically investigated. The *T_H_* was measured by a homemade device (Fig. S20). In Fig. 4B and Fig. S21, the *T_H_* values of water on uncoated steel and on the self-healing PSIB coating were −13.6 °C and −21.1 °C, respectively. This can be attributed to the decrease in the contact area of the water droplets on the surface, which is induced by the low surface energy of Si-based materials. After the addition of PDSB (5 wt%–15 wt%) into the PSIB matrix, *T_H_* decreased to −29.4 °C, which is twice that on the uncoated steel, indicating that ice nucleation on the AFP-inspired coating was markedly inhibited (Movie S6). As shown in Fig. 4C and Fig. S22, the water droplet on the uncoated steel began to freeze within 1 s of its contact with the surface, and it completely froze after 7s. In contrast, the complete frozen time of water droplets on the AFP-inspired coating was 339 s. Moreover, the ice propagation time of condensed water on the coatings was examined by an optical microscope coupled with a high-speed camera. As shown in Fig. 4D and E, and Fig. S23, the ice propagation rates were inversely proportional to the PDSB contents, and the ice propagation time on the AFP-inspired coating was 7,020 ms (propagation rate < 0.00048 cm^2^/s) (Movie S7). The results suggested that this coating can effectively inhibit ice propagation and diffusion, mainly attributed to the AFP-mimic PDSB.

Next, the deicing performance of the coatings was evaluated. The ice shear strength was measured to be 903.6 kPa (Fig. 4F) and 155.3 kPa on the steel surface and Si-based PSIB coating, respectively. After the addition of PDSB into the PSIB matrix, the ice shear strength was significantly decreased (38.9 kPa) because of the AFP-mimic property of PDSB. Over 20 icing–deicing cycles, the ice shear strength of the PPSB15 coating slightly fluctuated but remained lower than 50 kPa, which confirms its excellent stability (Fig. 4G).

### Self-healing properties of the anti-icing coating

**Fig. 5.**
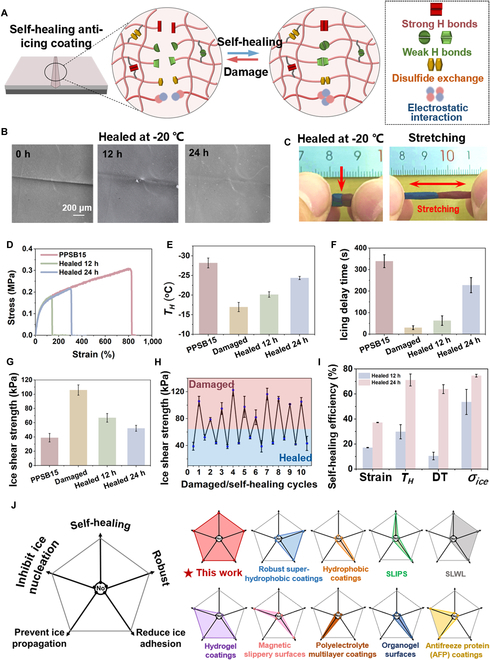
Self-healing properties of the AFP-inspired coating. (A) Autonomous self-healing mechanism of the coating, which is dependent on the synergistic interactions of multi-strength H bonds, disulfide metathesis, and electrostatic interactions between the zwitterionic segments. (B) Optical microscope images of the damaged PPSB15 coatings and its healing for 12 and 24 h at −20 °C. (C) Stretching of the PPSB15 film after self-healing for 12 and 24 h at −20 °C. (D) Stress–strain curves of the PPSB15 film healed for 12 and 24 h at −20 °C. (E) *T_H_*, (F) icing delay time, and (G) ice adhesion strength on the PPSB15, damaged, and healed coatings at −20 °C. (H) Ice adhesion strength of the PPSB15 coating after 10 damaging–healing and icing–deicing cycles. The coating is healed at room temperature. (I) Recovery efficiency of the PPSB15 coating healed at −20 °C. DT: icing delay time; *σ_ice_*: ice adhesion strength. (J) Comparison between the AFP-inspired coating and previously reported anti-icing coatings.

The self-healing performance of the PPSB15 anti-icing coating was investigated at room temperature and −20 °C. As shown in Fig. 5A, the supramolecular polymeric matrix can achieve fast autonomous self-healing as a result of the synergistic interactions of multi-strength H bonds, disulfide metathesis, and electrostatic interactions between the zwitterionic segments. At room temperature, the PPSB15 coatings can autonomously self-heal within 5 min (Fig. S24). After healing for 24 h at −20 °C, in which the injuries are commonly difficult to heal, the scratches on the PPSB15 surface completely disappeared (Fig. 5B). To evaluate the healing efficiency of a more serious injury, the PPSB15 specimen was cut into 2 pieces and put together into contact to heal at −20 °C. The healed sample exhibited a high stretchability and 37.1% healing efficiency (Fig. 5C and D).

In addition, the anti-icing and deicing properties of the healed PPSB15 coating were investigated. Figure 5E and Fig. S25 show that the *T_H_* of the damaged PPSB15 coating surface increased from −29.4 °C to −15.9 °C. After healing for 12 and 24 h at freezing temperature, *T_H_* decreased to −19.5 °C and −25.0 °C, respectively. The surface scratch was shallow and then disappeared, indicating the restored ability of the coating to inhibit ice nucleation. Figure 5F and Fig. S26 indicate that the icing delay time of the damaged coating decreased from 339 to 30 s and recovered to 228 s after self-healing at −20 °C. These results demonstrated the recovered anti-icing performance of the healed PPSB coating. The recovered deicing performance was then assessed (Fig. 5G and H). The ice adhesion strength of the damaged PPSB15 increased to 105.9 kPa and decreased to 52.2 kPa after self-healing, which is comparable to that (38.9 kPa) of the original coating. This indicates the inhibition of the formation of the ice–defect interlocking structure on this self-healing coating. Over 10 cycles of damaging–healing and icing–deicing (Fig. 5H), the defects on the coating were repeatedly and autonomously repaired and the deicing performance repeatedly recovered. Figure 5I illustrates the recovery efficiency of the PPSB15 coating, i.e., the strain (37.1%), *T_H_* (71.2%), icing delay time (63.8%), and ice adhesion strength (74.6%), after self-healing at −20 °C, indicating that the healed coating retained an anti-icing/deicing performance. The antifreeze self-healing of this coating contributed to the synergistic interaction of multiple dynamic bonds and lower glass transition temperature (*T_g_*). Thereinto, the lower S–S bond dissociation energy (60 kcal mol^−1^) of disulfide metathesis could efficiently improve the healing process and shorten the healing time at low temperature [26].

Outdoor anti-icing coatings would inevitably suffer from various complex environmental conditions such as ultraviolet irradiation, temperature variation, sandstorms, and acid rain erosion. Although natural AFPs exhibit superior antifreeze performance, they are more unstable than the AFP-mimic anti-icing coating when subjected to open air. These AFP-mimic coatings were subjected to various extreme conditions, including high temperature (100 °C), ultralow temperature (−196 °C), strong acid (pH = 0)/alkali (pH = 14), sand scouring, and ultraviolet exposure for 7 days. The surface morphology (Fig. S27A) and anti-icing/deicing test results (Fig. S27B and C) of the PPSB coating subjected to extreme conditions indicated its extraordinary durability, robustness, and stable anti-icing performance.

As shown in Fig. 5J, this AFP-mimic self-healing coating is superior to the anti-icing coatings reported in previous studies [9,13,33,45–51]. This coating exhibited high-efficient autonomous self-healing (5 min at room temperature and 24 h at −20 °C), as well as satisfactory anti-icing and deicing performance. Instead of fluorinated lubricants or artificial surficial structures used in currently advanced anti-icing coatings, both self-healing and anti-icing performance of this coating completely rely on the intrinsic properties of substrate materials, exhibiting the superiority for durability in large-scale practice applications. Moreover, the coating showed a high resistance to various extreme environments. In order to demonstrate its potential outdoor applications, PPSB15 coating was fabricated on a large-scale steel plate (30 cm × 20 cm) and commercial power cable (aluminum alloy, 50-cm long), respectively, as shown in Fig. S28, indicating that the raw materials and reactions can be scaled up for outdoor applications. Then, we applied this coating, compared with commercial anti-icing coating (ZS-611), on the power cable surfaces, which were placed in the simulated icing in rainy conditions at the Joint Laboratory of New Materials Application for Power Grid. Figure S29 shows that, compared with the uncoated cable, PPSB15 markedly reduces ice accretion by 20.5%. The ice accretion reduction of PPSB15 was 3.7 times less than that of the commercial anti-icing coating.

## Conclusion

In summary, a bioinspired self-healing anti-icing coating was developed to inhibit the surface-defect-induced icing processes. Defects can increase the absolute value of *E_ad_* of water molecules and accelerate heat transfer rate from the surface to air/water, resulting in preferential ice nucleation and propagation at the defects. Based on the understanding of key antifreeze sites in natural AFPs, a self-healing (at −20 °C) AFP-mimic-based anti-icing coating was developed. The coating exhibited outstanding anti-icing and deicing performance through the inhibition of ice nucleation, prevention of ice propagation, and reduction in ice adhesion, attributed to the intrinsic antifreeze properties of substrate materials instead of the commonly used artificial surface microstructure. Moreover, the coating exhibited a high resistance to various extreme conditions and an ability to operate outdoors. This work provides a high-efficiency, large-scale, and self-healing anti-icing coating for infrastructures in outdoor complex environments.

## Data Availability

The data are available from the authors upon a reasonable request.

## Supplementary Materials

Supplementary 1 Supplementary Information Figs. S1 to S29 Tables S1 and S2 Note S1
