# Supplementary material for: Inhibition of Defect-Induced Ice Nucleation, Propagation, and Adhesion by Bioinspired Self-Healing Anti-Icing Coatings
Source: Research (Wash D C). 2023 May 18;6:0140. doi: 10.34133/research.0140 (PMC10194051; doi:10.34133/research.0140)
Supplement: Supplementary 1 — Supplementary Information Figs. S1 to S29 Tables S1 and S2 Note S1 [file research.0140.f1.docx]

**Supporting Information to**

**I****nhibition of Defect-Induced Ice Nucleation, Propagation, and Adhesion by Bioinspired Self-Healing Anti-icing Coatings**

*Shu Tian ^1^, Ruiqi Li ^1^, Xinmeng Liu ^1^, Jiancheng Wang ^2^, Junyu Yu ^1^, Sijia Xu ^1^, Yunqing Tian ^1^, Jing Yang ^1, *^, Lei Zhang ^1, *^*

1 Department of Biochemical Engineering, Frontier Science Center for Synthetic Biology and Key Laboratory of Systems Bioengineering (MOE), School of Chemical Engineering and Technology, Tianjin University, Tianjin, 300350, China.

*2 Binzhou Institute of Technology, Weiqiao-UCAS Science and Technology Park, Binzhou City, Shandong Province, 256606, China.*

* Corresponding Author

E-mail: jing_yang@tju.edu.cn; lei_zhang@tju.edu.cn

**Table of Contents**

Materials and Methods……………………………………………………………2

Results and Discussion……………………………………………………………9

Supplementary Notes…………………………………………………………… 24

References………………………………………………………………… …….25

**Materials and Methods**

**1 Materials**

Dihydroxyl-terminated polydimethylsiloxane (HO-PDMS-OH, Mw=5600 g mol^-1^, from Dow Corning), vinylmethylsiloxane- dimethylsiloxane copolymer, trimethylsiloxy terminated (P(DMS-VMS), 800-1200 cSt, Mw=28000 g mol^-1^, from Gelest), Isophorene diisocyanate (IPDI, 99 %, from Nanjing Sheng Bicheng Chemical Technology Co., Ltd.), Dibutyltin dilaurate (DBTDL, 98%, from Adamas), 4,4′-dithiodianiline (SS, 98%, from Energy Chemical), 4,4′-bis(hydroxymethyl)-2,2′-bipyridine (BNB, 97%, from Shanghai UCHEM Biological Technology Co., Ltd.), 3-(dimethylamino)-1-propanethiol (DPT, 97%, from Beijing Ouhe Technology Co., Ltd.) and 1, 3-propyl sultone (1, 3-PS, 99%, from Shanghai Macklin Biochemical Co., Ltd), benzoin dimethyl ether (DMPA, 99%, from Nanjing Sheng Bicheng Chemical Technology Co. Ltd.) were all purchased without further purification. Solvents N, N′-dimethylacetamide (DMAc, 99.8%), tetrahydrofuran (THF, AR) and dichloromethane (CH_2_Cl_2_, AR) was purchased from Aladdin Biochemical Technology Co. LTD, Tianjin Kermel Chemical Reagent Co., Ltd. and Concord Technology, respectively. Commercial anti-icing coating ZS-611 was purchased from Beijing ZSWH Chemical Co., Ltd.

**2 Synthesis of** **AFP-inspired polymer**

The synthesis procedure of AFP-inspired polymer PDSB was described below (**Fig. S8a**). P(DMS-VMS) (5.00 g, 0.18 mmol), DPT (1.00/4.25 g, 8.40/35.7 mmol) and DMPA (0.03 g) were added to dried THF (15 mL) in a 50 mL flask, which was sealed. The reaction system was purged with Ar to eliminate oxygen and triggered by ultraviolet-irradiation at 365 nm at room temperature for 1 h. THF and excess DPT were removed by vacuum evaporation by 50 ^o^C to obtain light yellow viscous liquid P(DMS-DMA). The obtained P(DMS-DMA) (5.87/8.87 g, 0.18/0.18 mmol) was immediately dissolved to dried THF (15 mL) in a 50 mL flask and cooled to 0 ^o^C. A THF (5 mL) solution of 1,3- PS (1.00/4.36 g, 8.19/35.75 mmol) was dropwise added into the reaction system under vigorous stirring. After 24 h reaction at room temperature, product was collected and washed with dried acetone, and finally with diethyl ether. The obtained transparent solids were dried in vacuum at room temperature to get the PDSB The ^1^H NMR spectrum of PDSB was shown in **Fig. S8b, c**: ^1^H NMR (500 MHz, CDCl_3_) δ ppm: 3.26-3.53 (m, 4H), 3.10-3.18 (m, 6H),2.83-2.96 (m, 2H), 2.42-2.71 (s, 4H), 2.08-2.22 (dt, 2H), 1.90-2.07 (s, 2H), 1.90-2.07 (s, 2H), 0.05-0.30 (m).

**3 Synthesis of self-healing polymer**

According to the previous literature reported,[1] the self-healing polymer PDMS-SS-IB-BNB (PSIB) was synthesized (**Fig. S13**). SS, IB and BNB meant 4,4 '- thiodianiline, isophorone bisuria and 4,4' - bis (hydroxymethyl) - 2,2 '- bipyridine, respectively. HO-PDMS-OH (22.40 g, 4.00 mmol) in a 100 mL dried glass flask with a mechanical stirrer was heated at 100 ^o^C under vacuum (< 133 Pa) for 1 h for removing moisture, and then the reaction system was cooled to 70 ^o^C. IPDI (1.96 g, 8.00 mmol) and catalyst DBTDL (0.05 g) were dissolved in dry DMAc (15 mL), and added into the flask for 3 h under an Ar atmosphere. BNB (0.43 g, 2.00 mmol) and SS (0.50 g, 2.00 mmol) were further dissolved in dry DMAc (5 mL) and added into the reaction flask for another 3 h. After the reaction, the solution was put into a vacuum oven at 90 ^o^C for 24h to collect the product of PSIB polymer. The ^1^H NMR spectrum of PSIB was shown in **Fig. S13**, ^1^H NMR (500 MHz CDCl3) δ (ppm) 8.69-8.70 (d, 1H), 8.36–8.54 (s, 1H), 7.29–7.38 (s, 1H), 7.19–7.31 (s, 1H), 6.52–6.68 (dd, 1H), 4.14–4.28 (d, 2H), 3.58-3.72 (q, 2H), 3.40-3.48 (td, 2H), 2.89-3.10 (dd, 2H), 1.72-1.80 (d, 2H), 0.79-1.02 (m, 2H), 0.49–0.61 (m, 2H), 0–0.20 (s).

**4 Preparation of anti-icing/deicing coatings**

A certain amount of PSIB were dissolved to THF in 25 mL beaker. And PDSB dissolved in THF and CH_2_Cl_2_ (1/1, w/w) was added into above system and stirred for 30 min to achieve uniform mixing. The even mixture was coated onto steel or glass sheets and dried for 24 h at room temperature. Four ratio coatings were prepared and the proportions of each coating were shown in **Table S1**. PP and SB respresented PSIB and PDSB, respectively.The subscript number respresented the mass percent of PDSB polymer in PSIB.

**5 Characterization**

^1^H NMR spectra (Bruker, GER), fourier transform infrared spectroscopy (FTIR, Bruker, GER) and X-ray photoelectron spectroscopy (XPS, Thermo Fisher Scientific, USA) were used to characterize the chemical components and components of the synthetic polymers and coatings. Scanning electron microscope (SEM, FEI, USA),and energy dispersive spectroscopy (EDS, FEI, USA) were used to characterize the morphologies and elements distribution of the coating fracture surface. The glass transition temperatures (*T_g_*) and mechanical properties of the coatings were examined by differential scanning calorimeter (DSC, NETZSCH, GER) and universal testing machine (SUNS, China), respectively. The water dynamic contact angles were tested by optical θ meter (Powereach, China).

**6 Icing behavior of coating with defects**

Cryostage (LinKam, UK) and optical microscope (Nikon, JPN) coupled with a highspeed camera (Revealer, China) were used to research the icing behavior of coating with defects. The coating with defects was placed in a closed cell with a certain humidity and cooled to -15 ^o^C at a rate of 30 ^o^C/min. The process of water vapor condensation and water droplet icing on the coating surface was observed. Silver iodide solution was prepared according to previous literature, while the dispersive AgI nanoparticles were introduced ice nucleating agent. A droplet of AgI aqueous dispersion (1 μL) was placed atop the coating defect, and the coating cooled down at the rate of 5 ℃/min to observe the icing behavior.

**7 Computational Methods**

**7.1 Density functional theory calculation**

Density functional theory (DFT) calculation was used to concern the initial adsorption of water molecules onto the coatings with and without defect and was performed using the Cambridge Sequential Total Energy Package (CASTEP)[2, 3], and the exchange-correlation interactions were regarded by the generalized gradient approximation (GGA) in the Perdew-Burke-Ernzerhof (PBE) functional[4]. The all-electron potentials were pseudized with the Vanderbilt ultrasoft pseudopotential scheme, which formulated the electronic wave functions and potentials in an efficient way. We set the wave function in a plane-wave basis with an energy cutoff of 500 eV and used Monkhorst-Pack grids 5×5×1 to sample the two dimensional Brillouin zone. Set the force threshold and convergence criteria of the geometry optimization to 0.01 eV A^-1^ and 10 ^-5^, respectively. We used the conjugate gradient method to optimize the atomic positions. Silicone coatings without or with defects were simplified to signal silicone chains without or with Si-O bond broken. PDMS, PSBMA and PDSB copolymer were simplified to PDMS_2_, PSBMA_2_ and PD_1_SB_1_ as shown in **Fig. S10**.

**7.2 Finite element simulation**

Finite element (FEM) simulation was used to investigate the heat transfer of coatings to air and water. The 2-D model was established, in which triangular notches with a length of 2.5 μm and a height of 1 μm, 3 μm and 5 μm, respectively were introduced into the coatings. Selected silicone [solid] as the coating material and air/water as the material above the coating. The specific material properties were shown in Table S2. The physical fields of solid and fluid heat transfer were coupled, which the boundary conditions and initial conditions were defined to simulate the heat transfer on the coating. Initial conditions: the initial temperature of the coating was 253.15 K (- 20 ℃) and constant, and the initial temperature of water or air was 273.15 K (0 ℃). Boundary conditions: the temperature of coating-air/water two-phase interface was 253.15 K (0 ℃), and the surrounding boundary was thermally insulated.

**7.3 Molecular dynamics simulation**

Molecular dynamics (MD) simulations were carried out using the step of 2 fs with Gromacs-2019.6. The OPLS-AA force field with 1.20*CM5 atomic charge and TIP4P/Ice model were used for hydrophilic part DSB and water molecules. The Particle-Mesh Ewald (PME) method with the cutoff of 1.2 nm was used to describe the long-range electrostatic interaction, which the van der Waals interaction used the same cutoff. The starting conformation of SB was taken from a 10 ns equilibration of the macromolecule solvated in water at 300 K and 1 atm. A simulation box containing 8 free hydrophilic part SB molecules of equal mass and 1179 water molecules was established to simulate aqueous solution system. The simulation was run for 100 s with an isothermal-isobaric ensemble which the temperature was maintained at 258.15 K by a velocity-rescale thermostat with a coupling time of 0.1 ps and the pressure was maintained at 1 atm using Parrinello-Rahman method with a coupling time of 2.5 p. In addition, the density of the system was necessarily stabilized after a long enough pre-equilibration before the simulation. The properties of SB interaction energy with water were analyzed based on the mentioned simulation. To obtain the interaction energy for accuracy, OPC water model was used.

**8 Anti-freezing properties of PDSB**

**8.1 DSC test**

Differential scanning calorimetry (DSC, NETZSCH, GER) was used for researching the decreasing freezing point capacity of PDSB. 20 mg of DI water or PDSB aqueous solution in an aluminum pot was cooled to -40 ℃ at the rate of 10 ℃/min and kept for 2 min before heating to 20 ℃ at the rate of 2 ℃/min. The heat flux (W/g) change was measure, while the freezing point was the temperature when the solution began to melt.

**8.2 Nanoliter osmometer test**

Otago nanoliter osmometer (YASN, China) was used to investigate the effect of PDSB on the growth of single ice crystal. 50 nL DI water or PDSB aqueous solution was injected to a temperature-controlled sample holder containing silicone oil, which was cooled to -20 ^o^C until the solution completely frozen. And then heated the holder slowly till the single ice crystal appeared with the size remained within 20 s, and the temperature was the melting temperature (*T_m_*). The temperature was slowly reduced to a certain value value (*T_f_*). The supercooling temperature (*△T*=*T_m_*-*T_f_*) was 0.04 ^o^C in this work.

**8.3** **Ice recrystallization inhibition activity test**

Ice recrystallization inhibition (IRI) activity of DSB was performed by the “splat cooling method”. Dropped 12 μL DI water or PDSB aqueous solution into the quartz crucible placed on the cryostage (LinKam, UK), of which was precooled to -60 ^o^C. And then the temperature of the crucible was increased to -6 ^o^C at a heating rate of 20 ^o^C/min and annealed for 30 min. Subsequently, the images of the ice crystals were carried with a polarized optical microscope (Nikon, JPN). The mean largest grain size (MLGS) can be obtained by choosing and averaging ten largest single ice crystals in each image.

**9 Anti-icing/deicing properties of the coatings**

**9.1 Heterogenous ice nucleation test**

The heterogeneous ice nucleation temperature (*T_H_*) was measured by a homemade device (**Fig. S20a**). The coating was placed in the quartz crucible with a cover glass, while 1 μL water was dropped to the coating. The device was placed on the cryostage, which controlled by a program was cooled to -30 ^o^C at the rate of 5 ^o^C/min. The heterogeneous ice nucleation (HIN) temperature (*T_H_*) of the coating was defined by the temperature where the water droplet converted from transparent to opaque within 1 s under the optical microscope[5]. More than 10 freezing events for each coating were measured to obtain the average value of the *T_H_*.

**9.2 Icing delay test**

Icing delay time (DT) of the surface was performed using optical θ meter (Powereach, China) with a cooling stage (JINGIE, China). The stage was precooled to -15 ^o^C and Ar gas was pumped into the specimen chamber for the dry environment. Water was dropped on the surface and the droplet changes were recorded by a high-speed CCD camera under the continuous shooting mode till the water droplet was fully frozen and icing delay time was also obtained. The data of DT represented the average value of more than 5 freezing events for each surface.

**9.3 Ice propagation test**

The ice propagation time of condensed water on different coatings were tested by an optical microscope coupled with a highspeed camera as shown in **Fig. S20b**. The water droplets condensed on the coating in the closed quartz crucible with a cover glass via “evaporation and condensation processes” and two water droplets (0.5 μL) were dropped at the edge of the coatings. The quartz crucible was placed on the cryostage, which was heated to 50 ^o^C to make water droplets evaporate firstly. And then the cryostage was cooled to -20 ^o^C at the rate of 1 ^o^C/min. The ice propagation process that the transparency of vaporized droplets changed gradually was recorded by the highspeed camera.

**9.4 Ice adhesion test**

Ice adhesion strength was measured by a homemade device which was consisted with a cooling stage coupled with a force transducer (Imada ZP-50 N/ 500 N, JPN) as shown in **Fig. S20c**. The coating was positioned on the cooling stage and a hollow curette was placed on the coating, and 450 μL water was injected into the curette. The temperature of the cooling stage was cooled to -15 ^o^C and held 4 h. Ar gas was pumped into the specimen chamber for the dry environment during the whole process. The force transducer was installed on a motion stage to push the cuvette at the rate of 0.1 mm/s. The maximum force was recorded for calculating the ice adhesion strength of the coating. We obtained the the average value from three samples.

**10 Self-healing properties of the coatings**

Samples for surface self-healing tests were prepared by using a knife to make scratches on the coating surface. The damaged coating was placed at - 20 ℃ or room temperature to heal the defects for 12 and 24 hours, and then the scratch morphology, HIN temperature and ice adhesion strength were investigated, respectively. Samples for self-healing efficiency tests were prepared by dissolving the coating materials in THF/CH_2_Cl_2_ (w/w, 1/1). Subsequently, removed bobbles in the solution by ultrasonic treatment and poured the solution into a PTFE mold. The solution which evaporated at room temperature overnight. The obtained sample was further dried at 65 ^o^C in a vacuum oven for 24 h and cut into a rectangular shape specimen with dimension of 20 × 2.5× 1 mm^3^. Cut the specimen into two pieces and put them together into contact for healing at -20 ^o^C. Mechanical tensile-stress tests were performed before and after healed to evaluate the self-healing efficiency of the coating, which was defined as the ratio of healed fracture strain relative to the original fracture strain.

**11 Extreme environment-resistance properties of the coatings**

The coatings were placed on the 100 ^o^C heating platform for 60 min; put into liquid nitrogen for 10 min; poured by 2 L HCl solution (pH=0), 2 L NaOH solution (pH=14); soured by 2 L quartz sand; radiated by 40 W UV lamp for 7 days, respectively. The HIN temperature and ice adhesion strength of the coatings were measured and the surface morphologies were characterized by SEM.

**Results and Discussion**


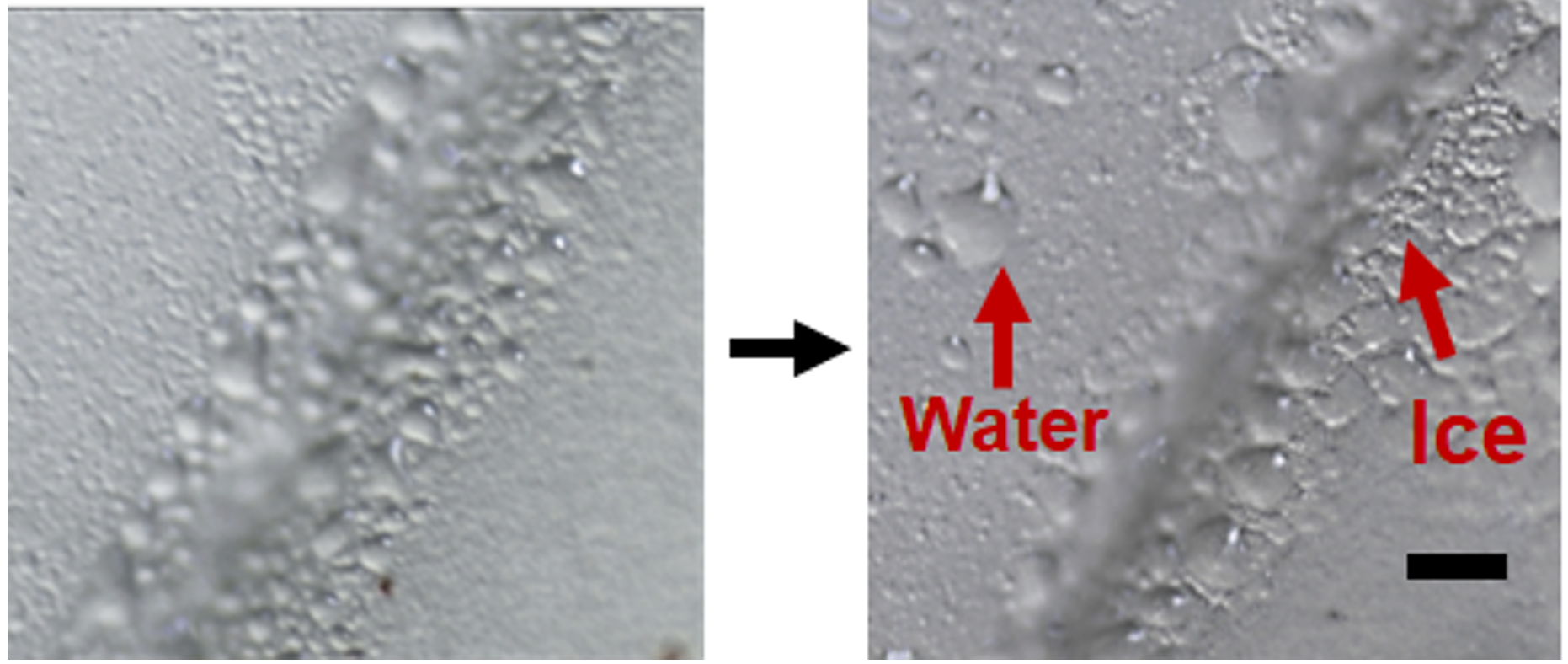


Fig. S1 Condensation and freezing of water vapor on the coating with defect.


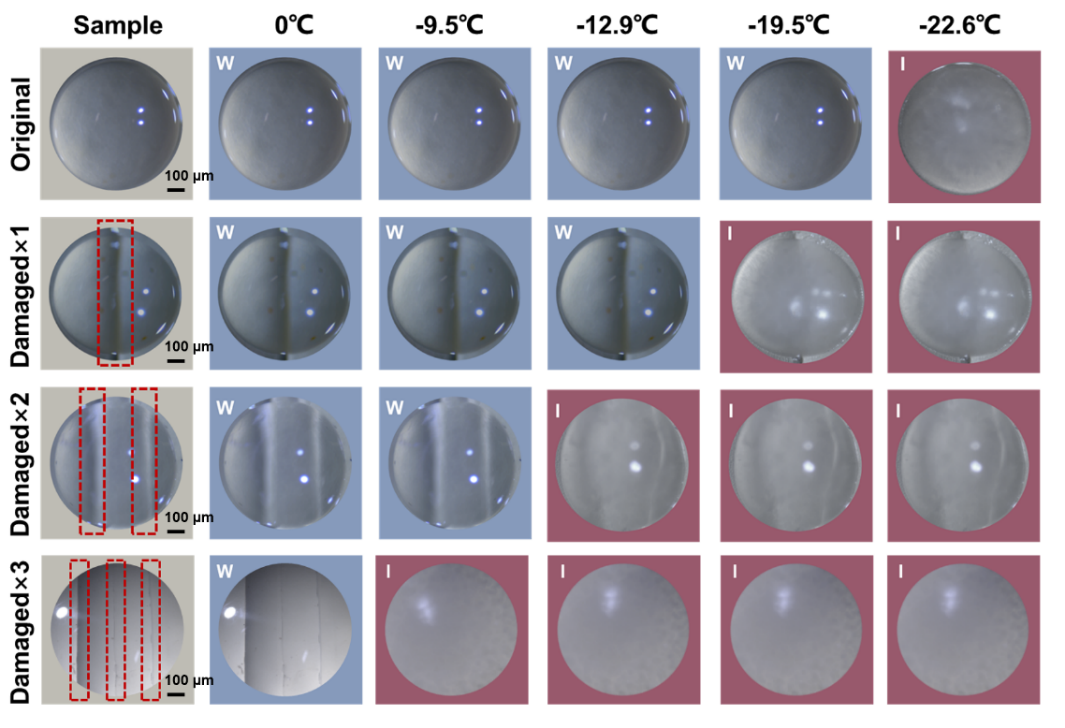


Fig. S2 Optical microscopic images of water droplet freezing at different temperatures of silicone coatings without and with 1-3 defects.


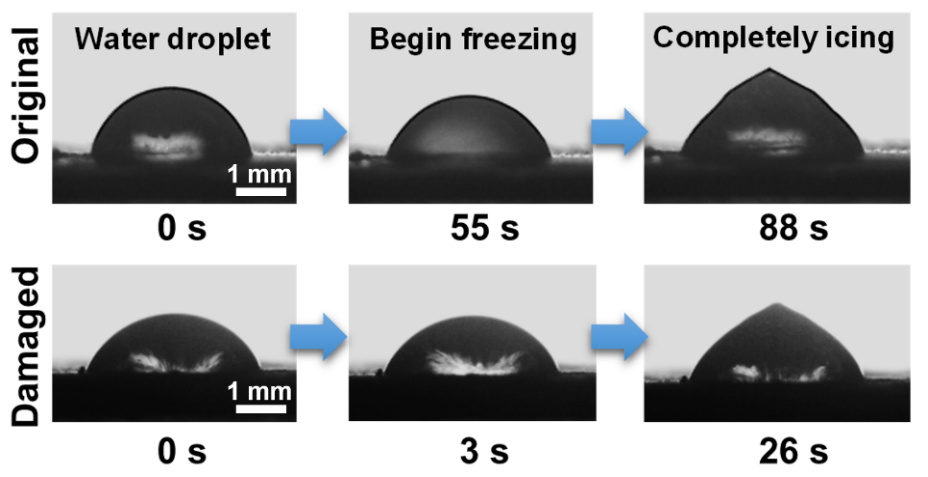


Fig. S3 Optical microscopic images of water droplet freezing at -15 ^o^C on the coatings without or with defect.


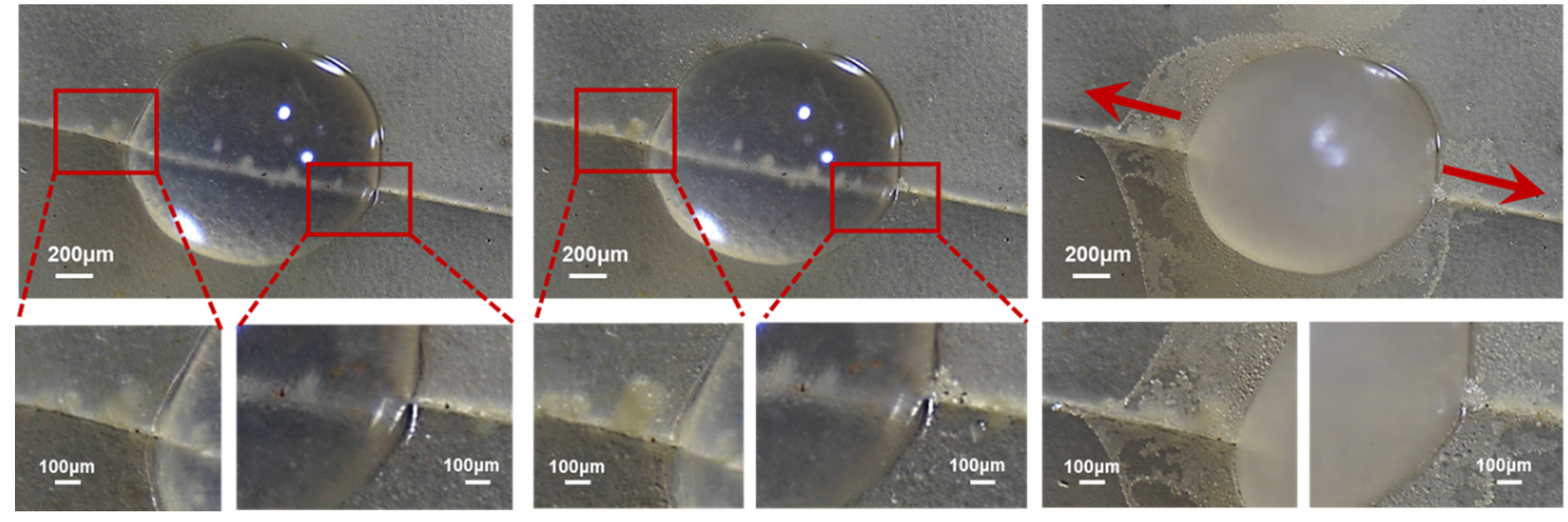


Fig. S4 Freezing process of the water droplet with nucleating agent on the defective coating.


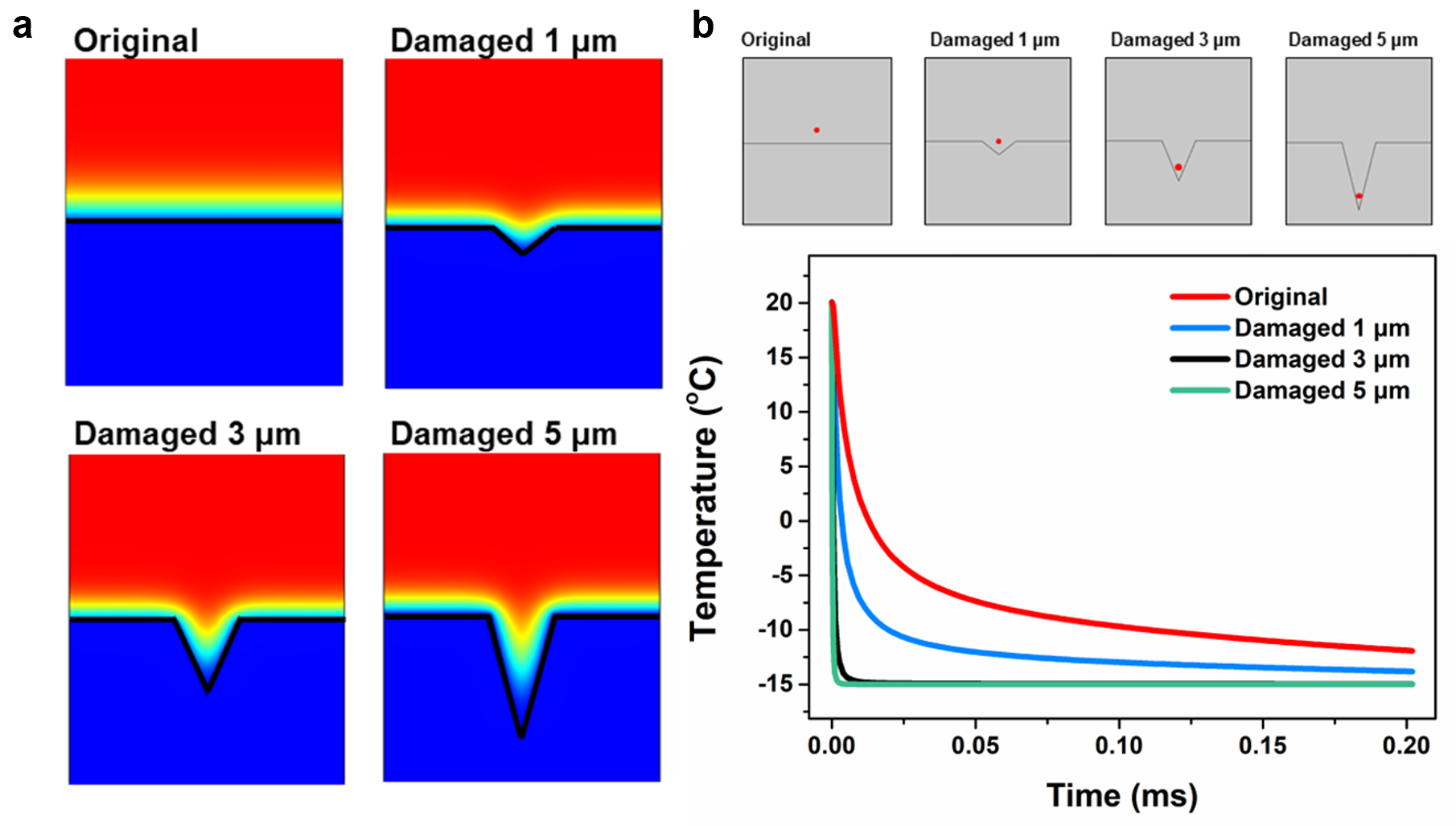


Fig. S5 Heat transfer model and time-temperature curve of coating-air system. The coatings with different depth defects of 1, 3, 5 μm.


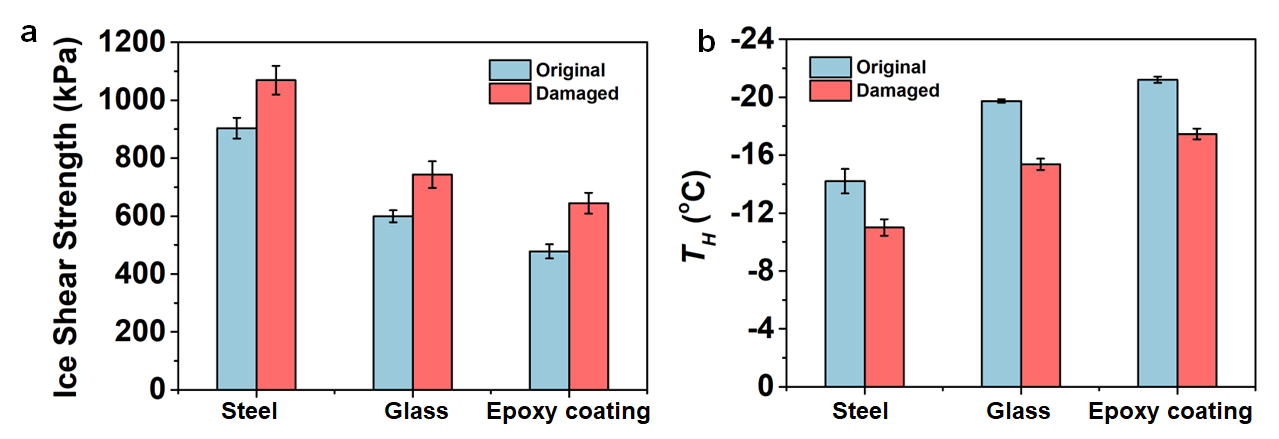


Figure S6 (a) HIN temperature and (b) ice shear force of steel glass and epoxy coating without and with defects.


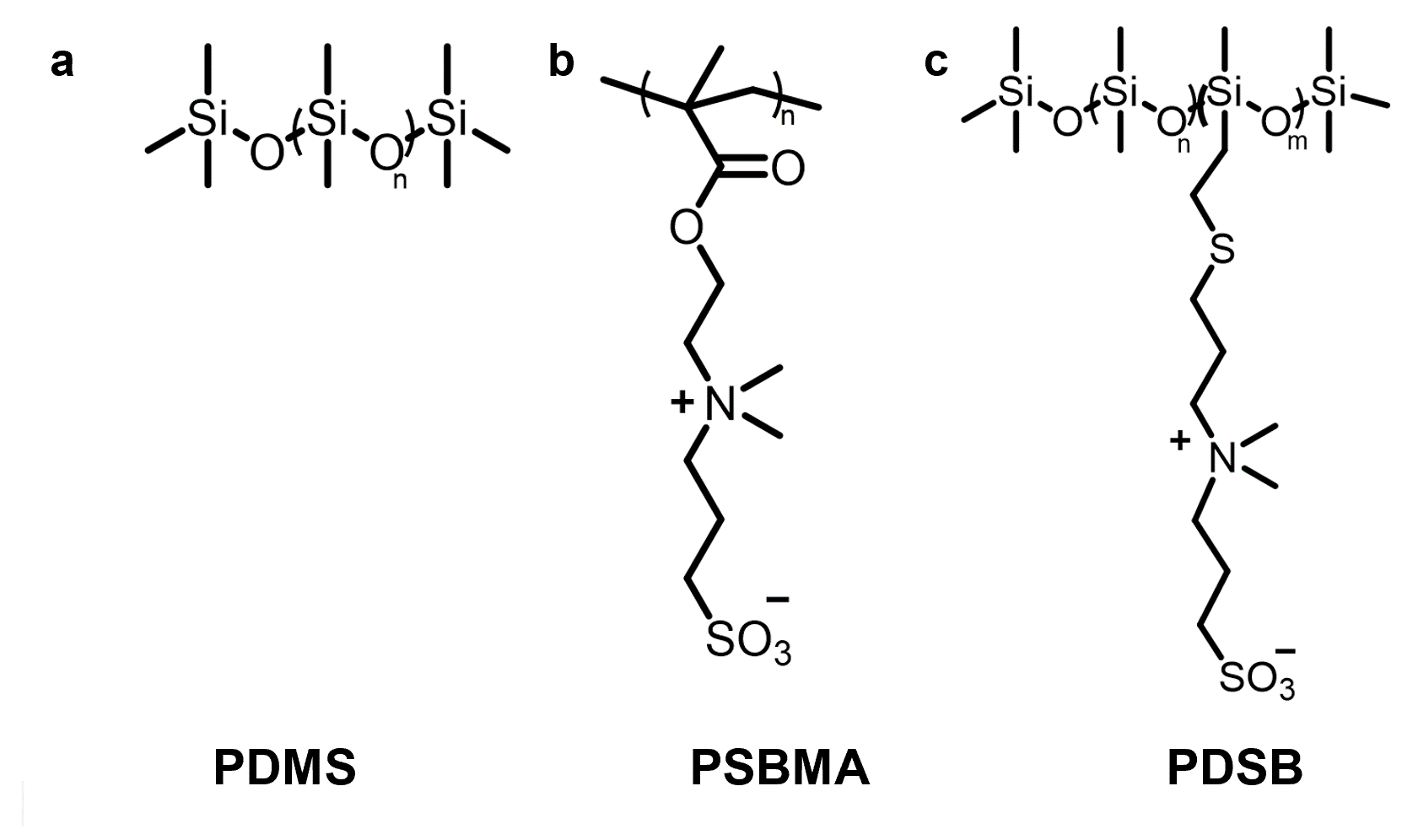


Fig. S7 Molecular structure of PDMS, PSBMA and PDSB.


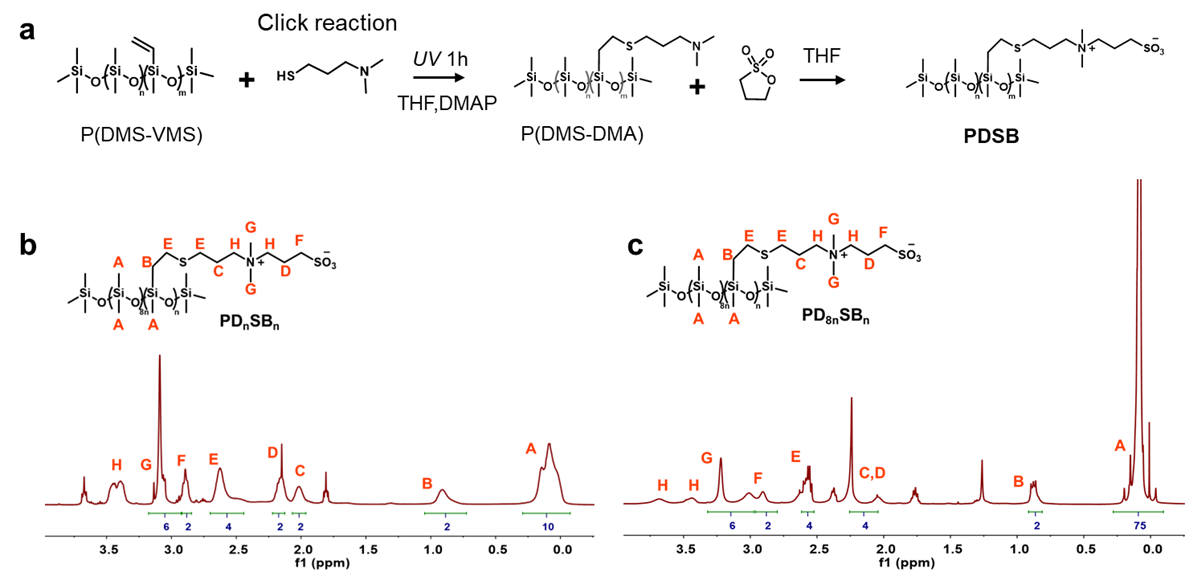


Fig. S8 (a) Synthesis process of AFPs mimetics copolymer PDSB. 1 H NMR spectra of (b) PDnSBn and (c) PD_8n_SB_n_.


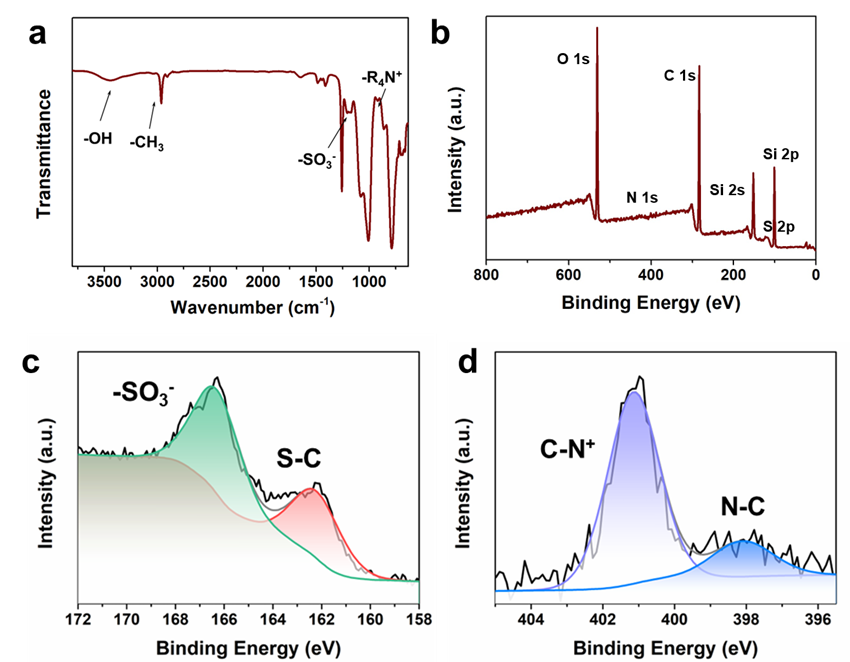


Fig. S9 (a) FTIR spectra of PDSB. (b) Wide region, (c) S 2p and (d) N 1s XPS spectra of PDSB.

As shown in Fig. S9 (a), there were absorption peaks at 2963 cm^-1^, 1198 cm^-1^ and 921 cm^-1^, indicating hydrophobic (methyl), negative (sulfonate) and positive (quaternary amine) groups that corresponding to the relevance amino acid residues of AFPs, respectively.[6, 7]

The XPS spectra of PDSB showed that the S 2p spectra of PDSB can be divided into peaks of -SO^3-^ (169.5 eV) and S-C (164.9 eV), as well as the N 1s spectra of PDSB was divided into C-N^+^ (402.1 eV) and N-C (399.1 eV), respectively.[8, 9]


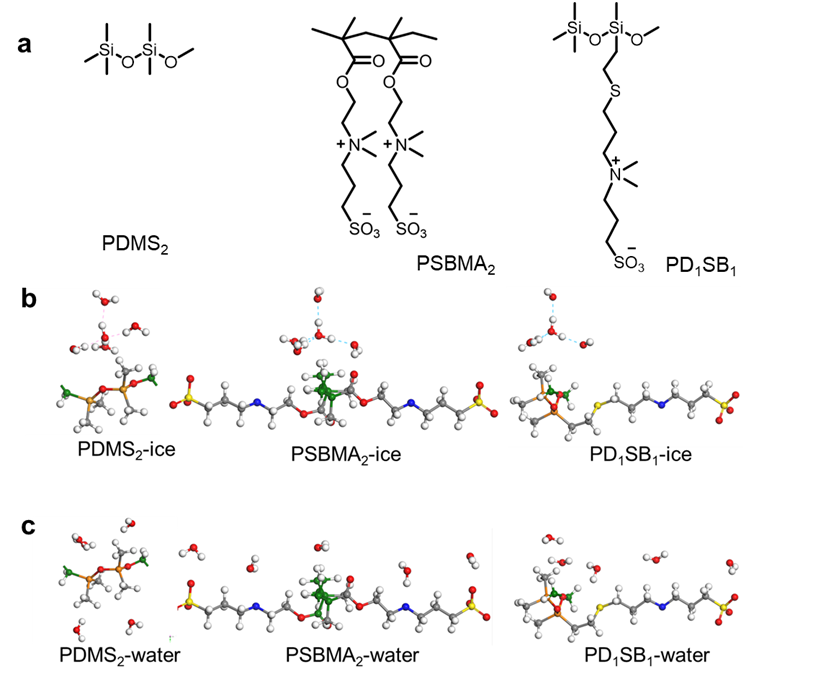


Fig. S10 (a) Molecular structure of PDMS_2_, PSBMA_2_ and PD_1_SB_1_. (b, c) Adsorption models for ice and water molecules on PDMS_2_, PSBMA_2_ and PD_1_SB_1_ chain.


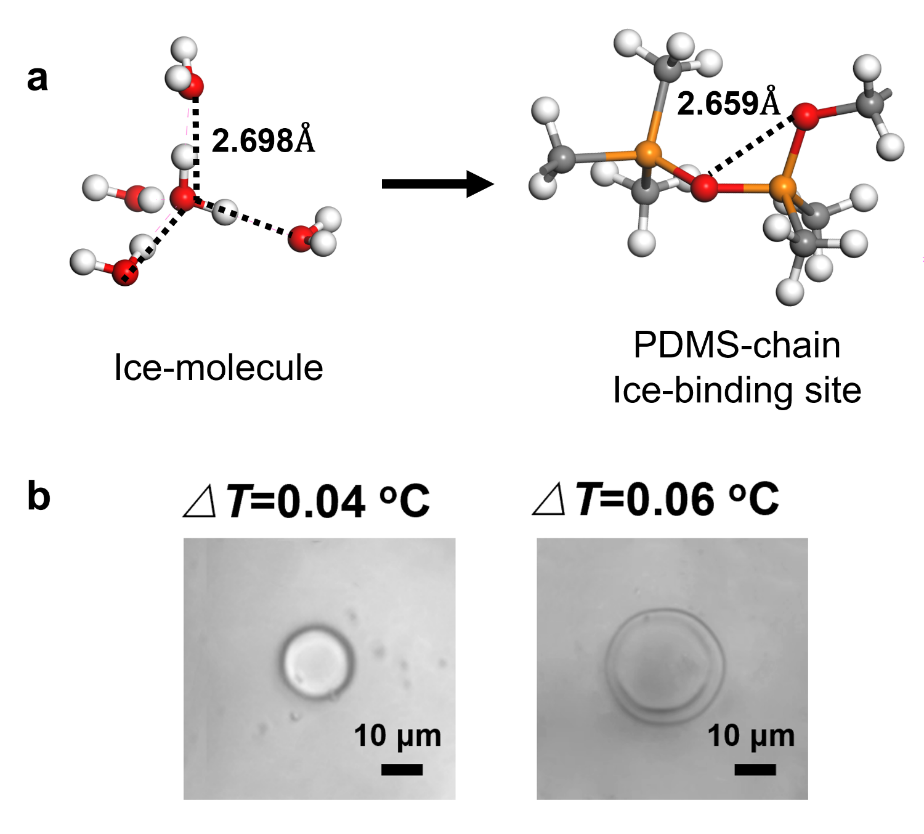


Fig. S11 (a) Pattern matching of PDMS chain to the the lattice spacing of ice. (b) A single ice crystal growth in PDSB solution under *△T*= 0.04℃ and 0.06℃.


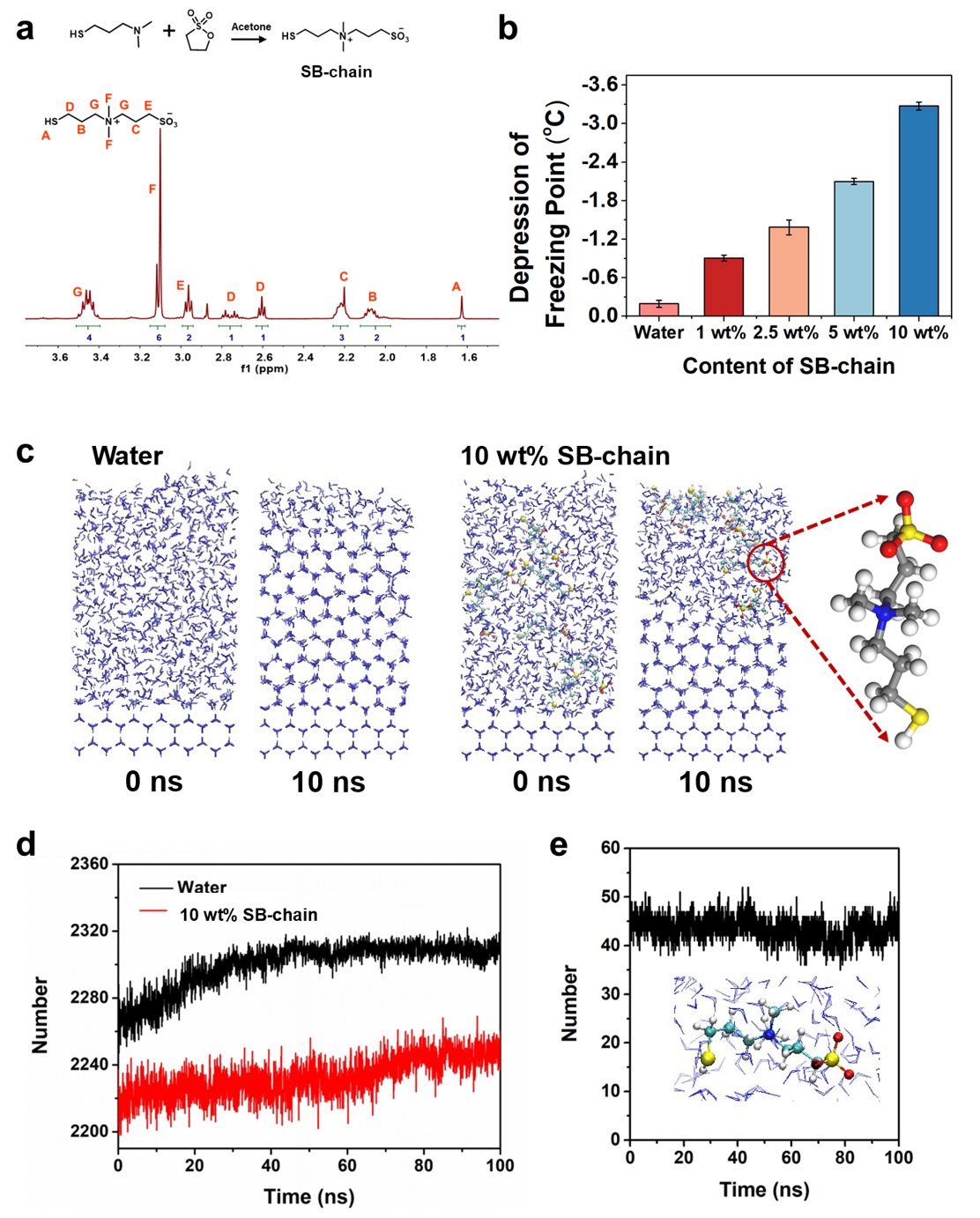


Fig. S12 (a) Synthesis process and ^1^H NMR spectra of hydrophilic part DSB of AFP-inspired materials PDSB. ^1^H NMR (500 MHz CDCl3) δ (ppm) 3.40-3.51 (m, 4H), 3.08-3.15 (d, 6H), 2.94-2.99 (td, 2H), 2.57-2.82 (t, 2H), 2.18-2.26 (m, 3H), 1.97-2.12 (dd, 2H), 1.61-1.65 (s, 1H).

(b) Freezing point of water and SB aqueous solution with different concentrations. (c) Snapshots of ice-water system without or with SB-chain at t =0 ns and 10 ns. Growth kinetics of H-bonds between (d) water molecules, (e) water and DSB molecules.

Molecular dynamics (MD) simulation was used to illustrate the effects of SB on ice formation at molecular level. As shown in **Fig. S12c**, The SB-chain segment molecule was modeled and a SB-water system was established in MD; an unrestricted 10-ns MD simulation showed that SB-water system still had non-freezing water, and SB chains could be evenly dispersed in liquid phase after 10 ns regardless of its initial position. The water molecules in the pure water system without SB segments were completely frozen into ice crystal molecular after 10 ns. Further, by calculating number of H-bonds between water molecules, water and SB molecules, it was shown that SB segments can bind to water molecules through their ionic solvation effects and H bonds (Fig. S12d, e), which reduce the water freezing point and inhibit ice nucleation.


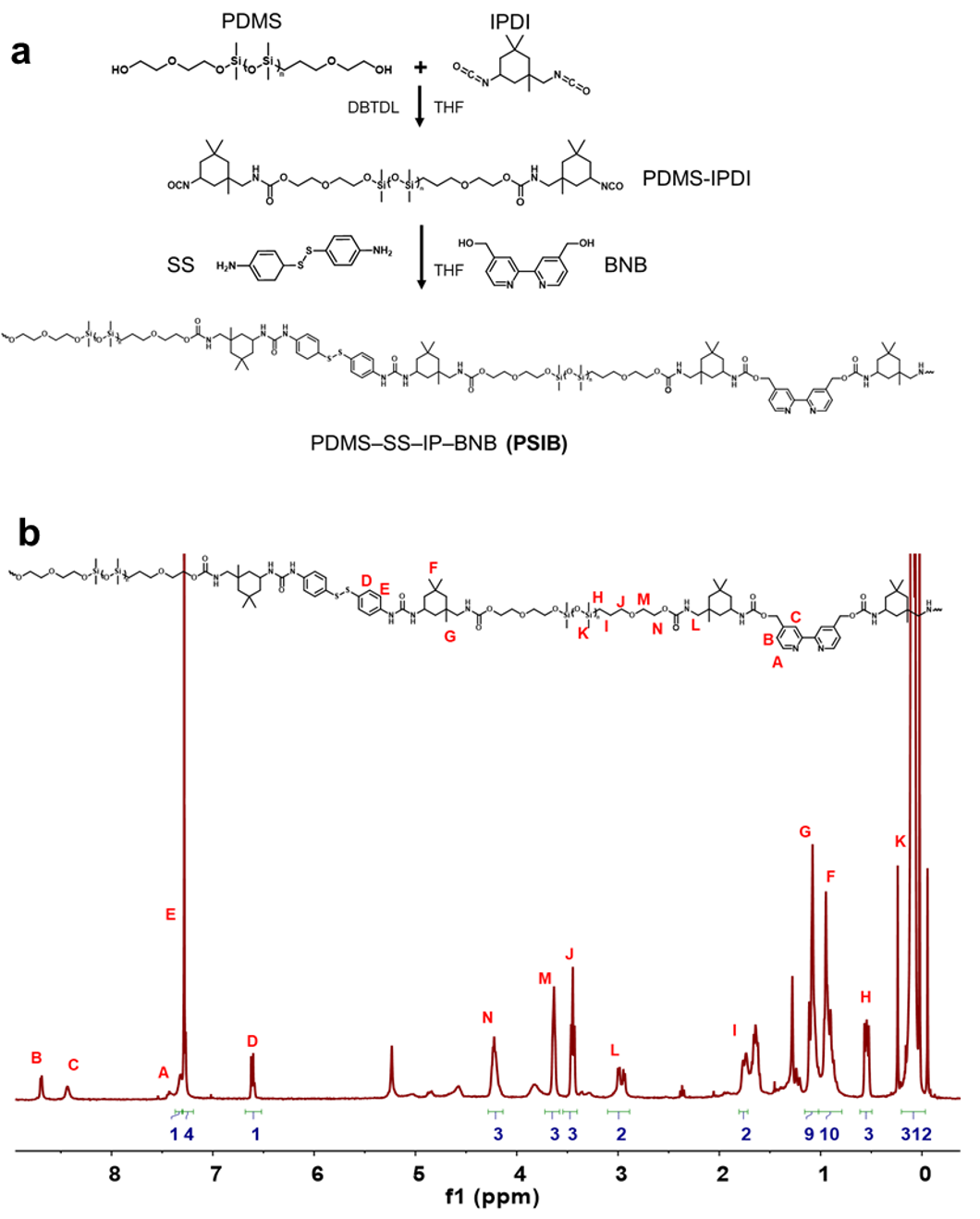


Fig. S13 (a) Synthesis process of self-healing polymer PDMS-SS-IB-BNB (PSIB). (b) ^1^ H NMR spectra of PSIB.

**Table S1** The proportions of each coating.

| **Sample** | **PSIB (g)** | **PDSB (g)** | **THF (mL)** | **CH_2_Cl_2_(mL)** |
| --- | --- | --- | --- | --- |
| **PSIB** | 2 | 0 | 2 | 2 |
| **PPSB5** | 2 | 0.1 | 2 | 2 |
| **PPSB10** | 2 | 0.2 | 2 | 2 |
| **PPSB15** | 2 | 0.3 | 2 | 2 |


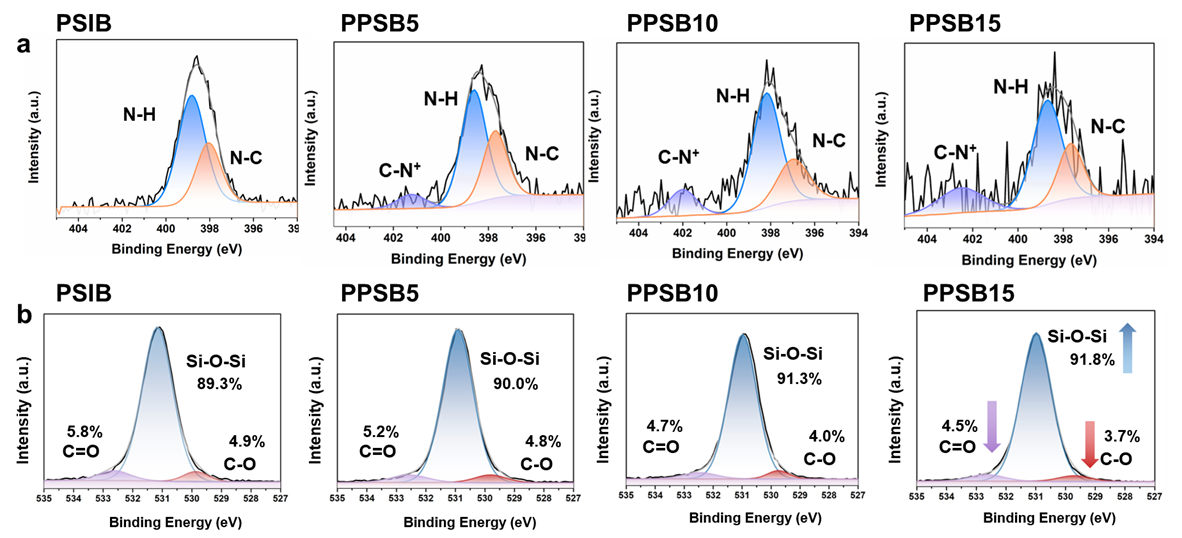


Fig. S14 (a)N 1s and (b) O 1s XPS spectra of PSIB, PPSB5, PPSB10 and PPSB15.

Firstly, the compositions of the anti-icing coatings were characterized by XPS. **Fig. S14a** showed the the N 1s spectrum of pure self-healing coating PSIB and composite coatings with different content of PDSB. For PSIB coating, N 1s spectra only can divided into two peaks of N-H (399.4 eV) and N-C (399.1 eV), and the characteristic peak of C-N^+^ appeared at 402.1 eV after adding PDSB. And with the increase of PDSB content, the intensity of C-N^+^ peak was higher, indicated the increase of NIBS on the surface of PSIB coating.

Furthermore, the O 1s spectrum of different coatings can divide into three peaks of C=O (533.7 eV), Si-O (532.6 eV) and C-O (531.7 eV) as shown in **Fig. S14b**. The results showed that the intensity of Si-O-Si was increased with the addition of PDSB, meanwhile the IBS content of the coating surface was also increased.


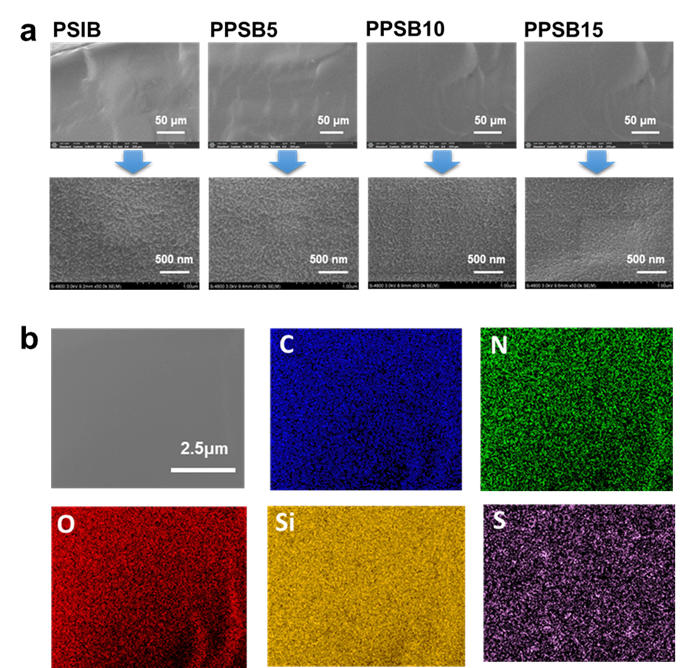


Fig. S15 (a) Fracture surface SEM and (b) element mapping images of different coatings.

Amphiphilic materials are generally prone to phase separation, which will decrease the functionality and homogeneity of coatings. The fracture surface SEM images (**Fig.S15a**) showed that the coatings exhibited no obvious phase separation after adding amphiphilic PDSB into the hydrophobic PSIB.

The elemental mapping images of PPSB15 coating in **Fig. S15b** also showed the homogeneous distribution of elements.


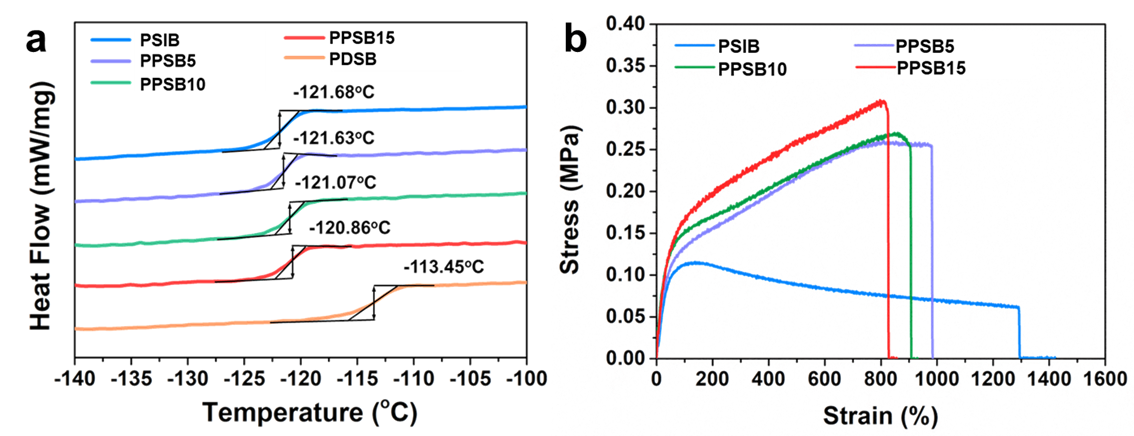


Fig. S16 (a) DSC thermograms and (b) representative tensile stress-strain curves of the coatings.

We measured the glass transition temperature (*T_g_*) of the coatings by DSC. As shown in **Fig. S16a**, the addition of PDSB increased the *T_g_* of PSIB coating, so as to prove the formation of interpenetrating polymer network (IPN) structure in the coatings. As shown in **Fig. S16b**, the increase of the fracture strength after adding PDSB also can illustrate the IPN structure of the composite coatings. The formation of the IPN structure further proved that the hydrophobic PSIB maintained uniform after adding the amphiphilic PDSB, and there was no obvious phase separation.[10, 11]


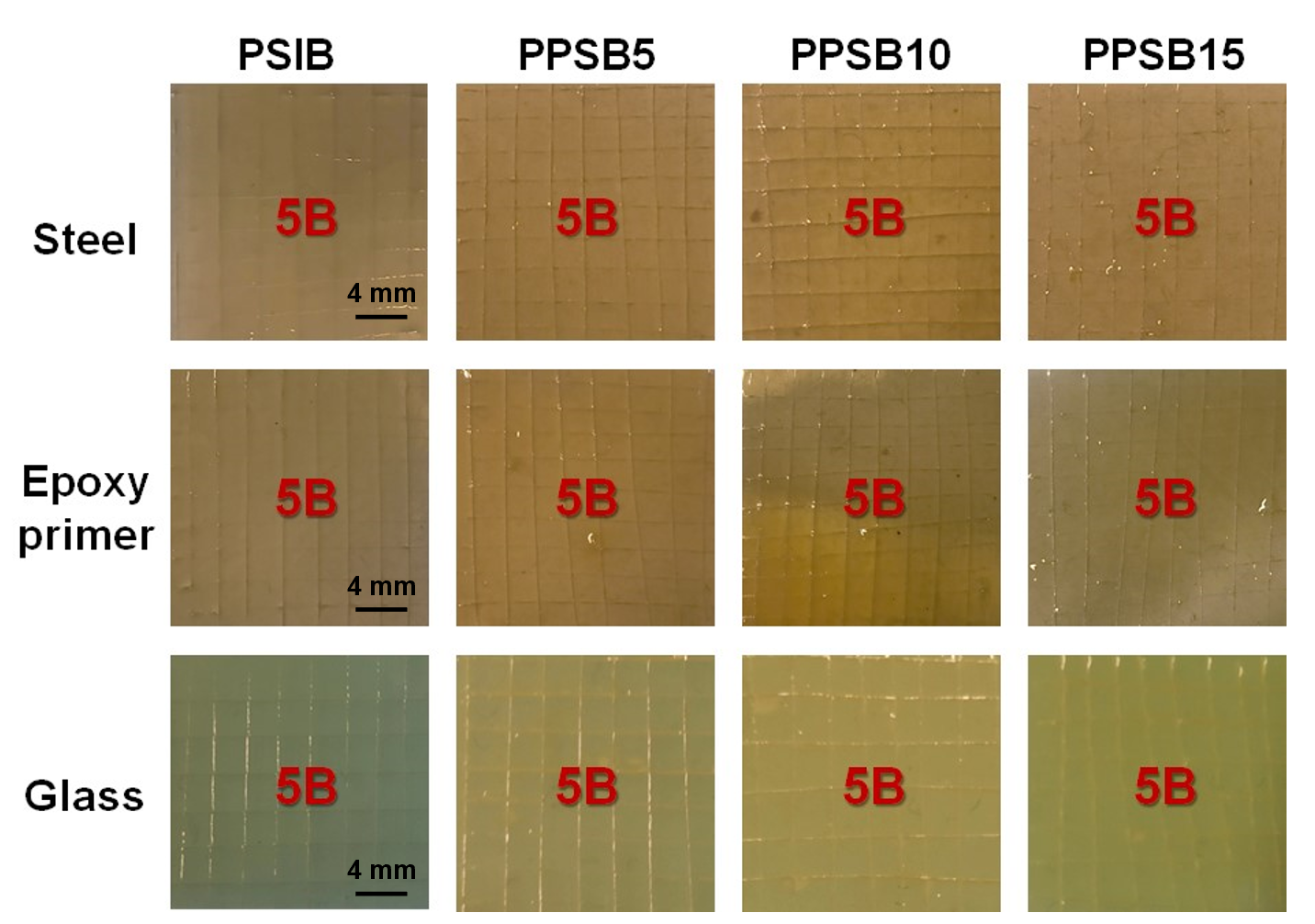


Fig. S17 The photographs of the coatings after crosshatch adhesion tests.


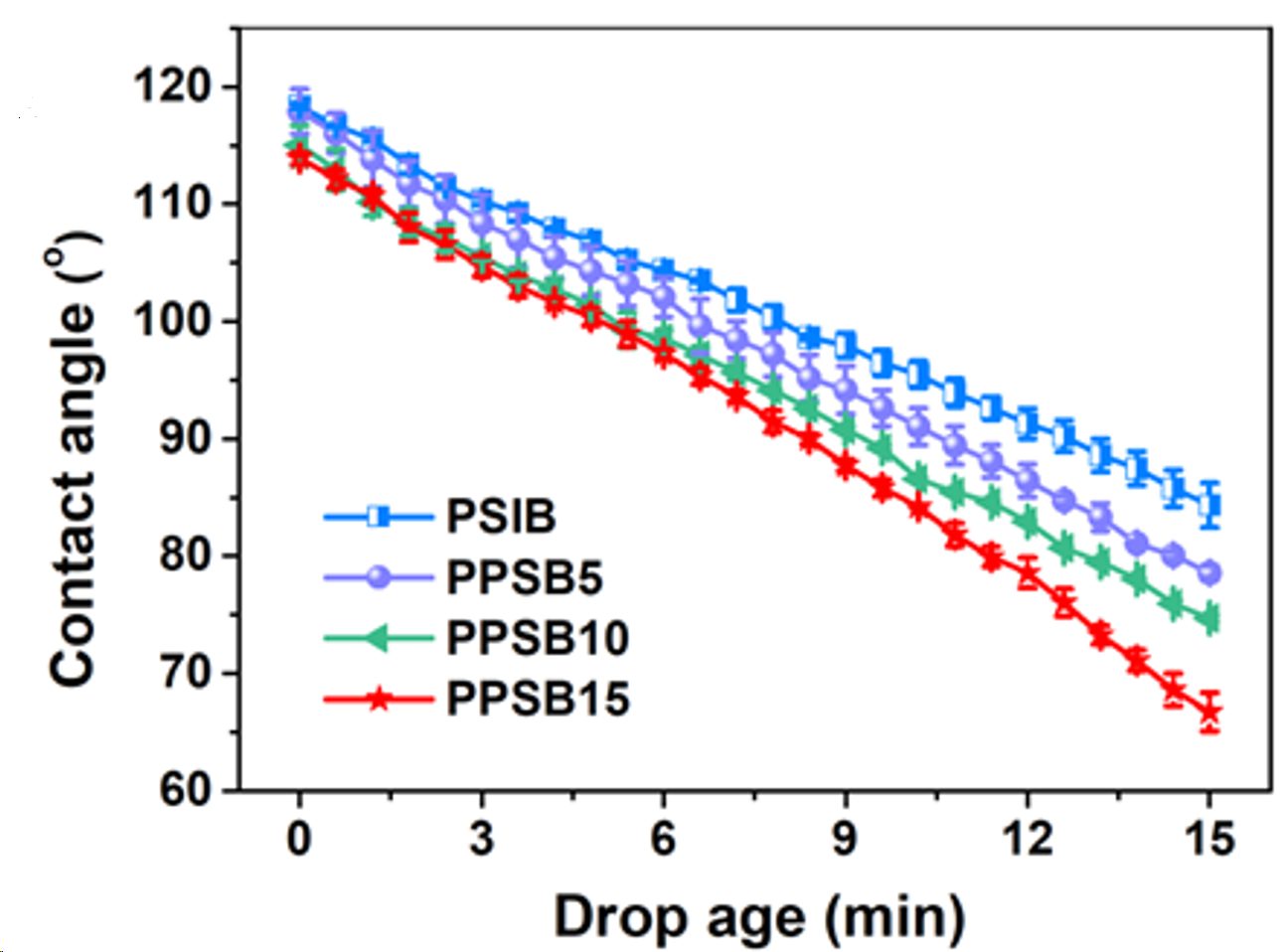


Fig. S18 The water dynamic contact angle of the coatings.


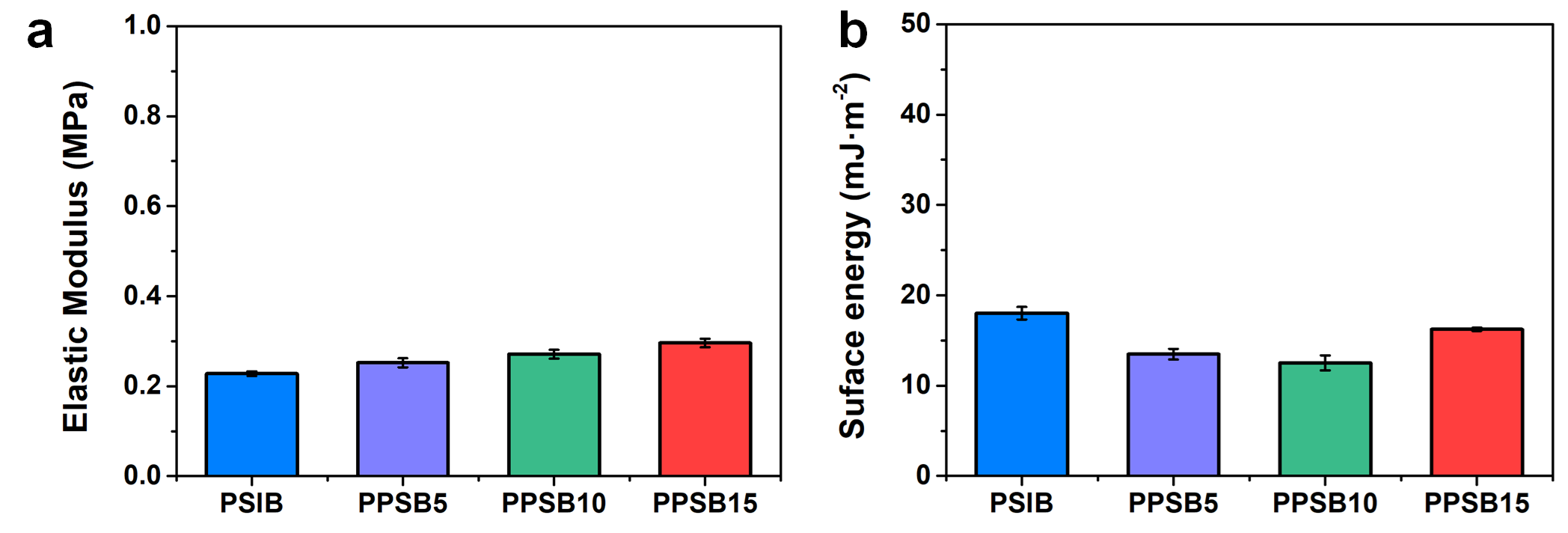


Fig. S19 (a)Elastic modulus and (b) Surface energy of the coatings.


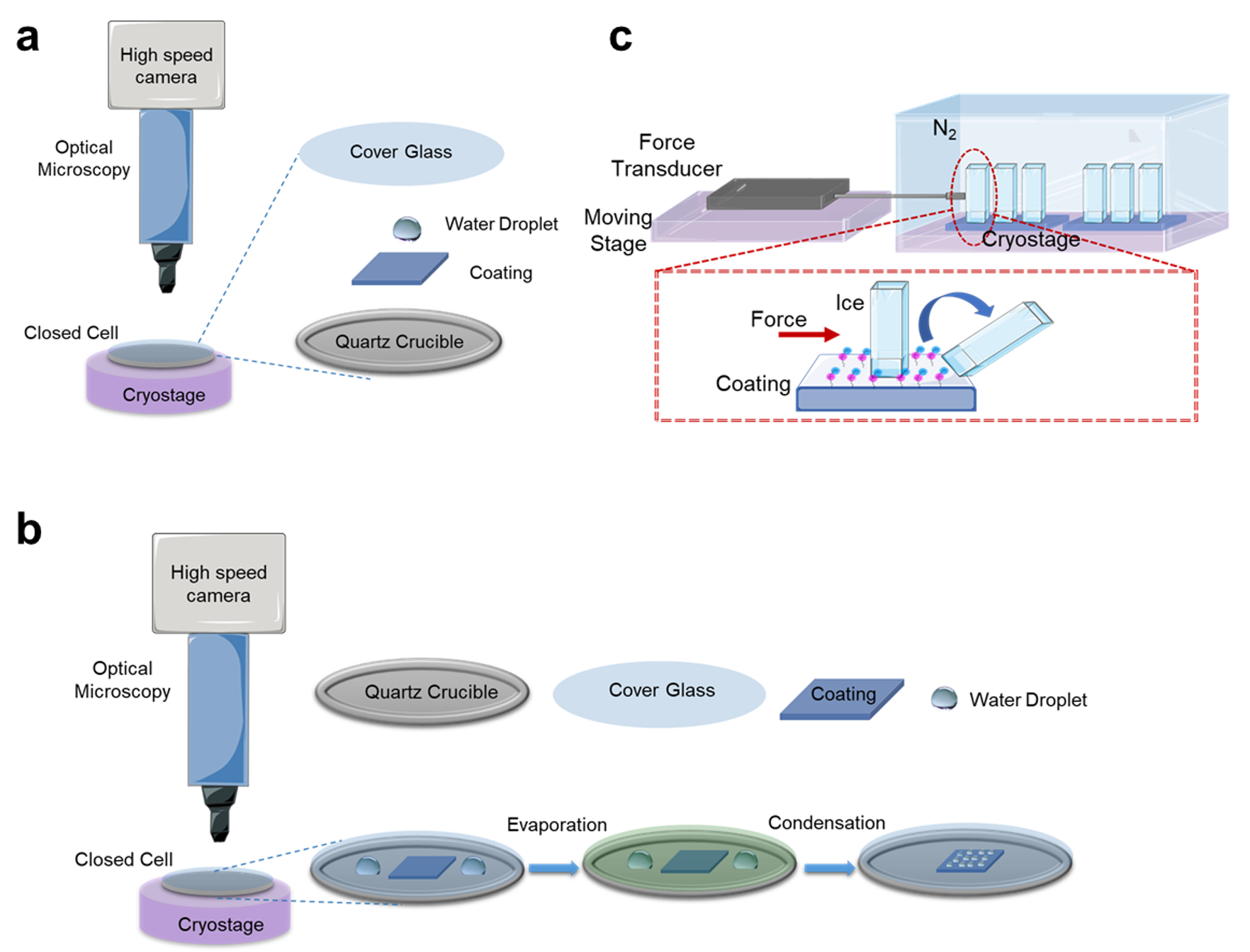


Fig. S20 (a) Homemade device used to measure the HIN on coatings. (b) The device equipped with an optical microscope and a highspeed camera used to measure ice propagation rate on coatings. (c) Schematic diagram of the device for ice adhesion strength measurement.


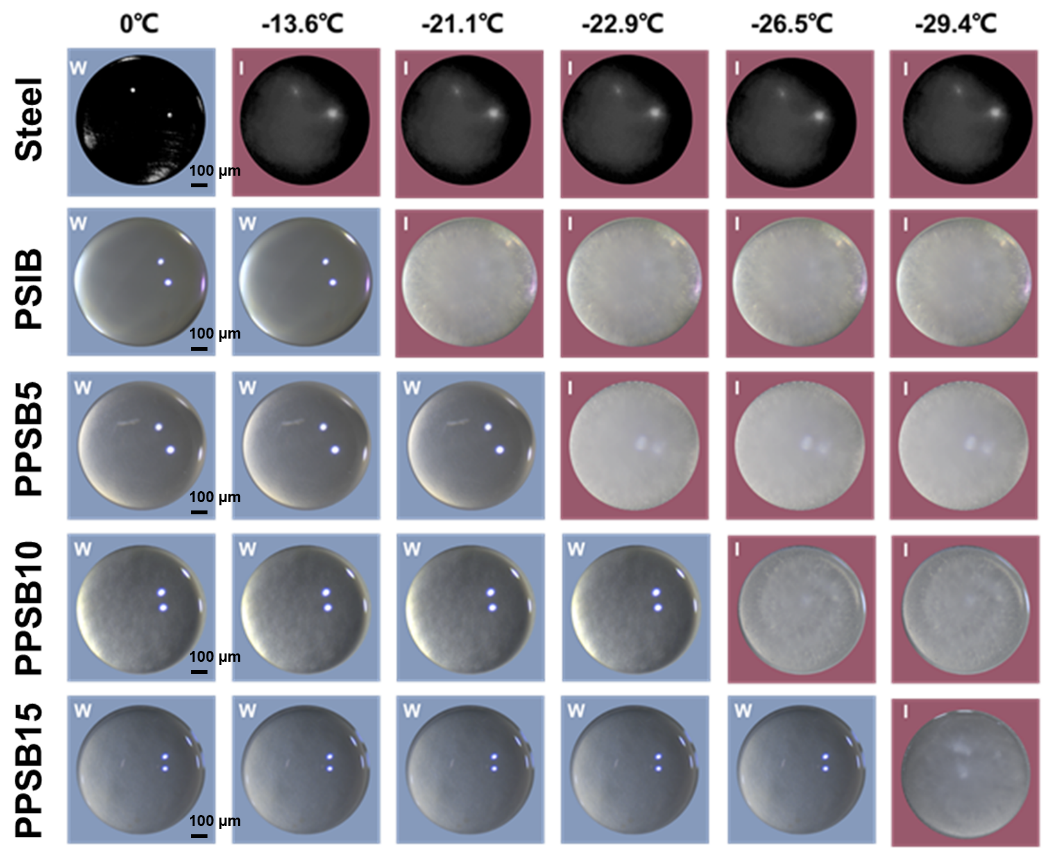


Fig. S21 Optical microscopic images of water droplet freezing at different temperatures on different coatings.


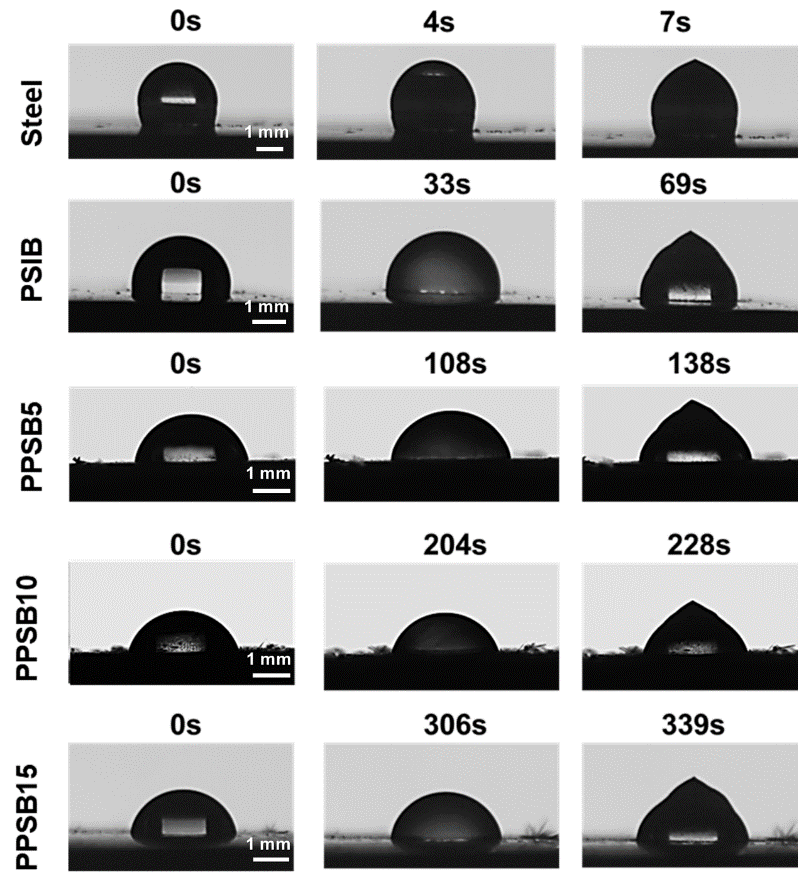


Fig. S22 Optical microscopic images of water droplet freezing at -15 ^o^C on different coatings.


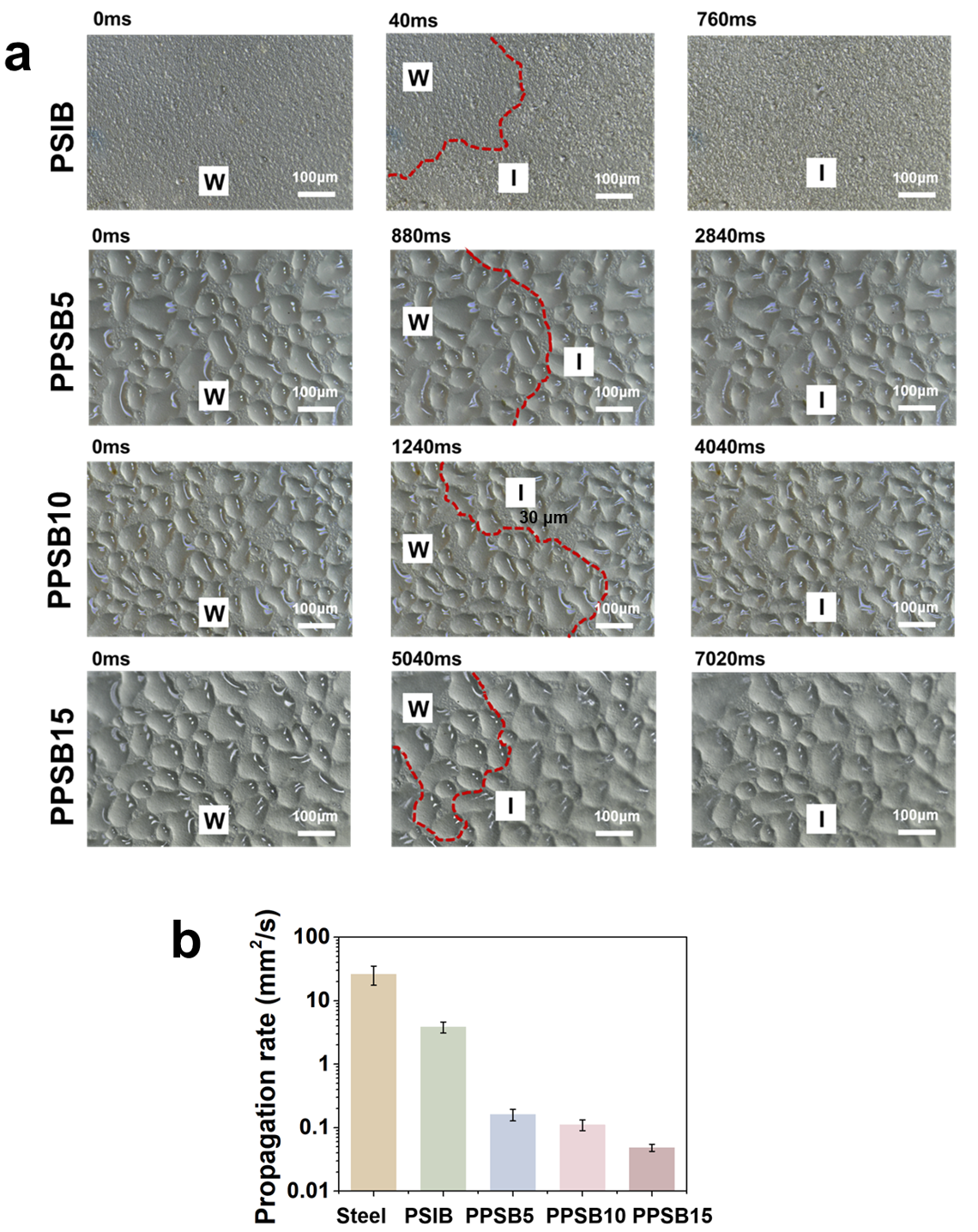


Fig. S23 (a) Time-resolved optical microscopic images of ice propagation on the different coatings. (b) Change in the propagation rate on different coatings.


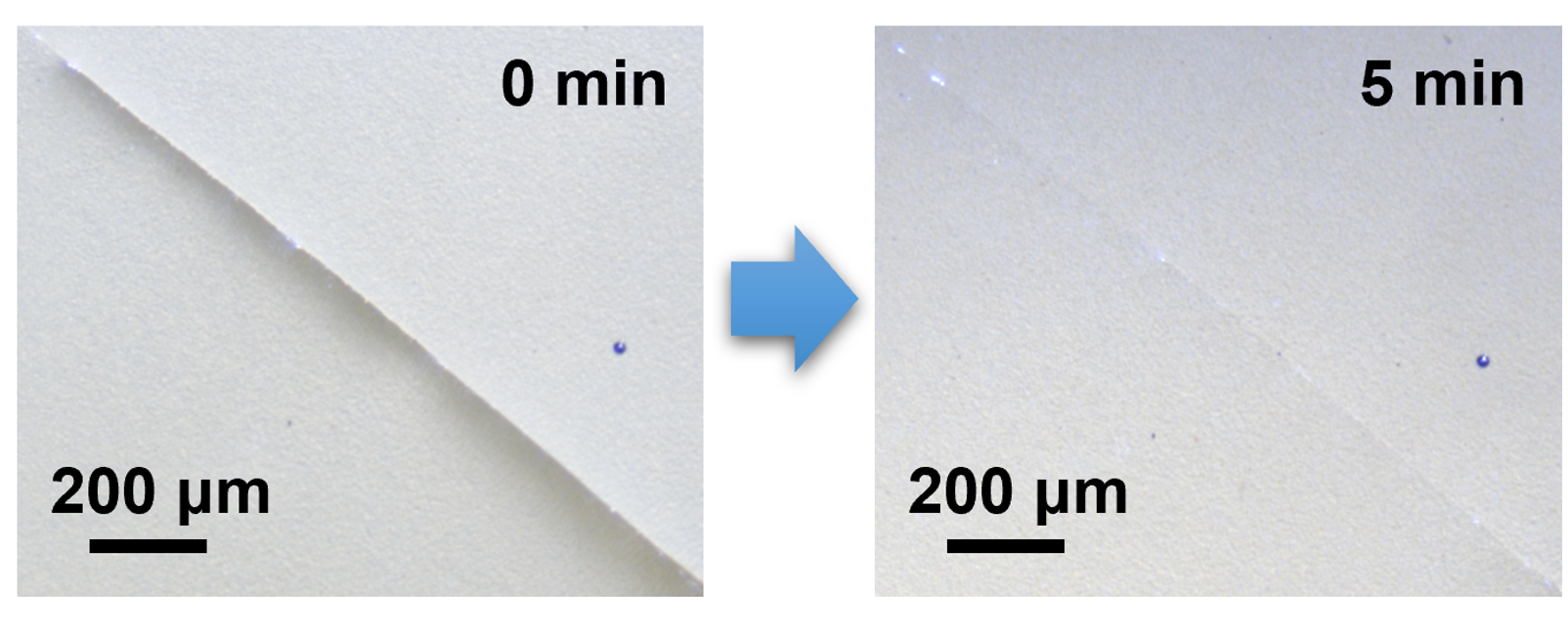


Fig. 24 Optical microscope images of the damaged PPSB15 coatings and its healing at room temperature.


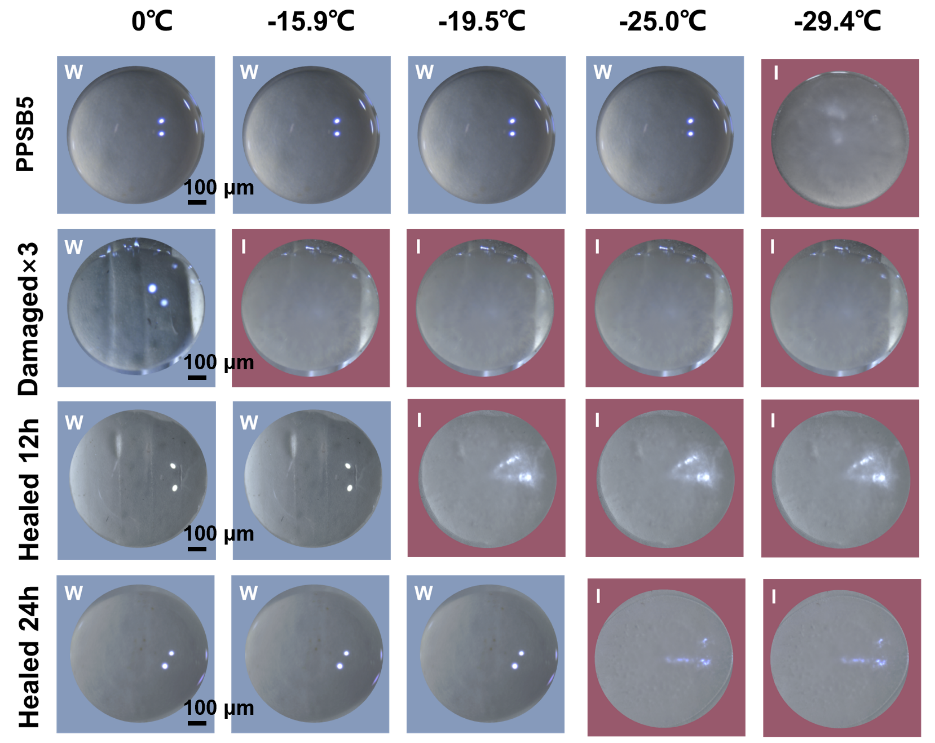


Fig. S25 Optical microscopic images of water droplet freezing on damaged and healed PPSB15 coatings.


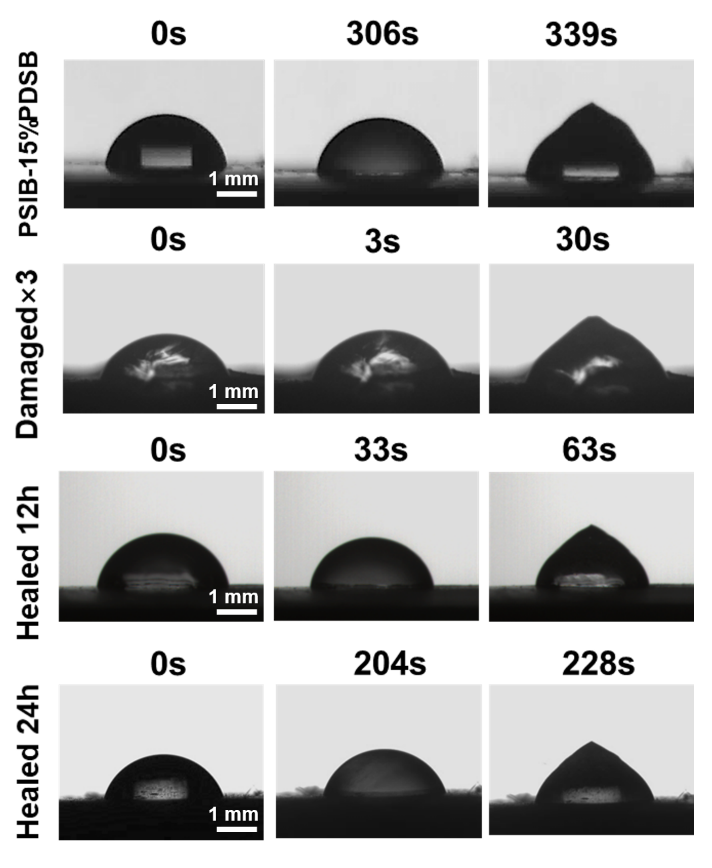


Fig. 26 Optical microscopic images of water droplet freezing at -15 ^o^C on damaged and healed PPSB15 coatings.


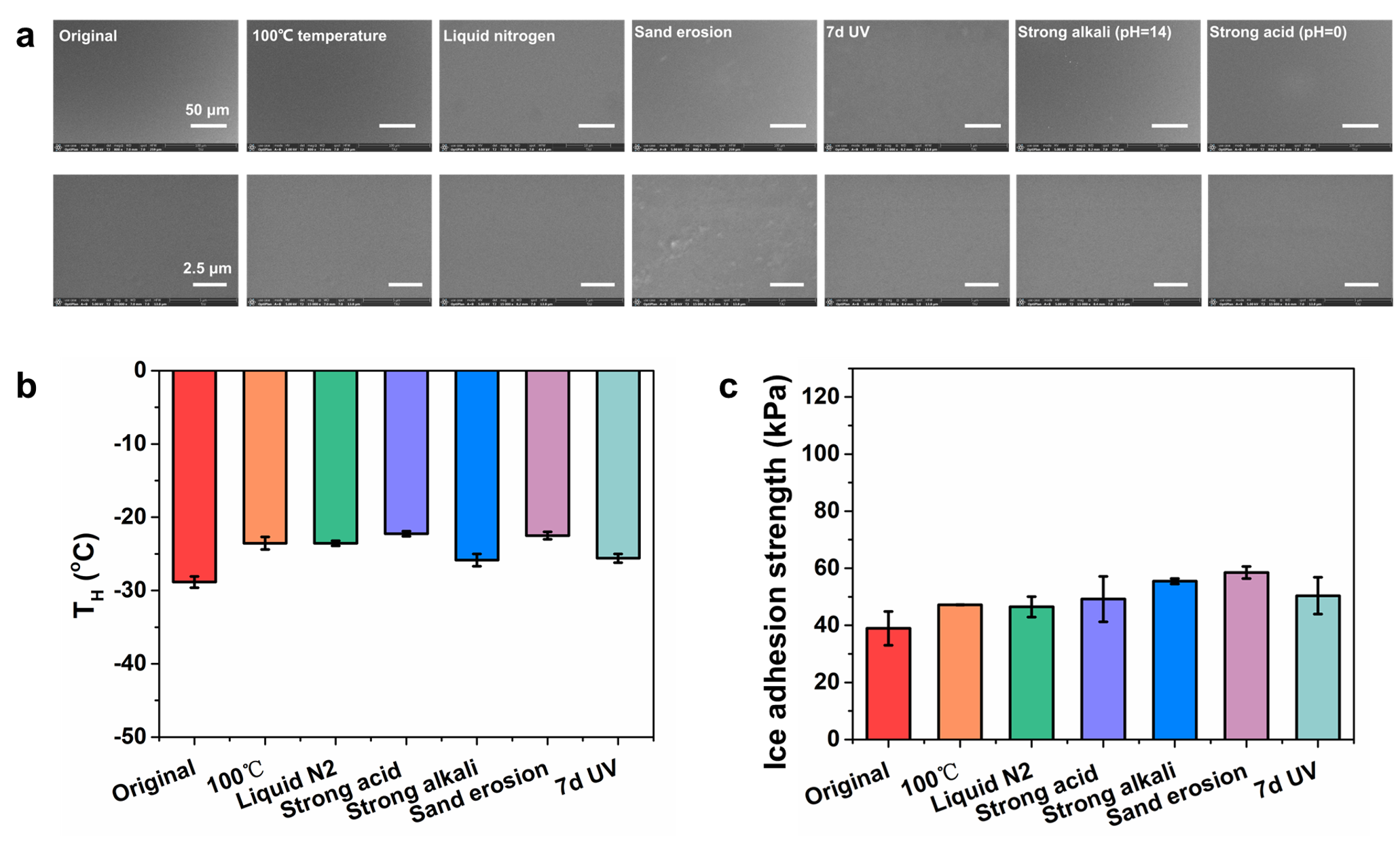


Fig. S27 (a) SEM images, (b) HIN temperature and (c) ice adhesion strength of PPSB15 coating surface after the experience of different extreme environments.


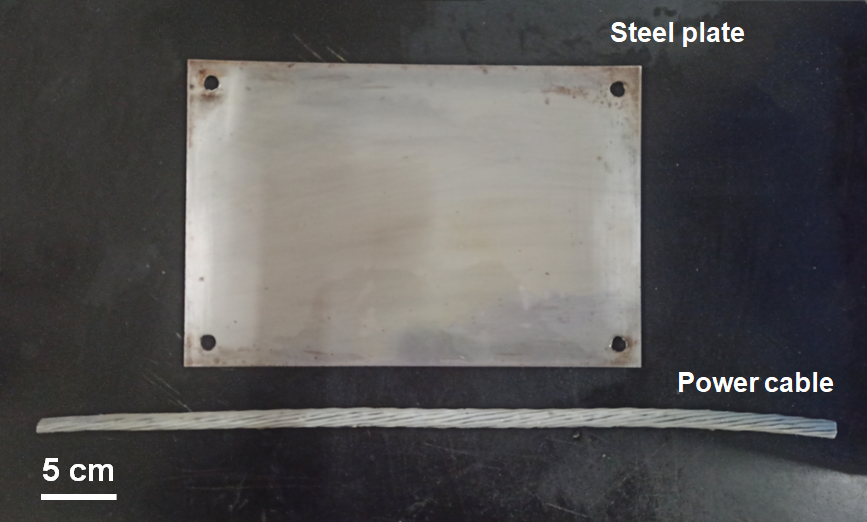


Fig. S28 PPSB15 -coated 30 cm × 20 cm steel plate and 50 cm long power cable.


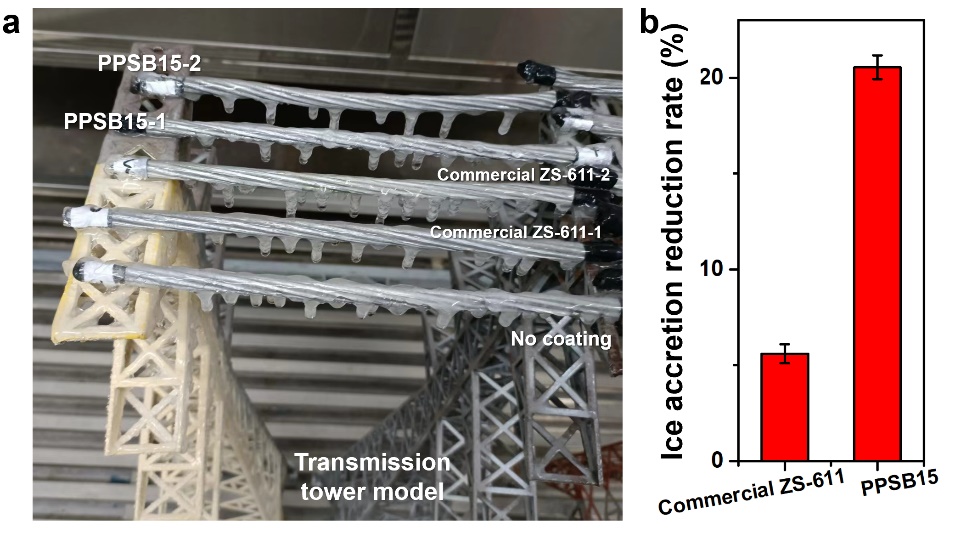


Fig. S29 (a) Simulated icing test of PPSB15 and commercial ZS-611 coated cables on the transmission tower model. (b) Ice accretion reduction rate of commercial ZS-611 and PPSB15 coated cables compared with uncoated cable.

Table S2 Weight and ice cover rate of cables after simulated icing test

| **Sample** | **Original weight/g** | **Final weight/g** | **Ice accretion/%** | **Ice accretion reduction/%** |
| --- | --- | --- | --- | --- |
| **PPSB15-1** | 22.6 | 36.9 | 63.3 | 21.2 |
| **PPSB15-2** | 22.7 | 37.3 | 64.3 | 19.9 |
| **Commercial ZS-611-1** | 22.7 | 40.0 | 76.2 | 5.1 |
| **Commercial-ZS-611-2** | 22.4 | 39.3 | 75.4 | 6.1 |
| **Uncoated cable** | 21.8 | 39.3 | 80.3 | - |

**Supplementary Notes**

**Supplenmentary Note 1.** Various previous works have related ice adhesion strength (τ) of elastic surfaces with the Kendall equation[12]:

τ∝$\sqrt{W_{a}G/t}$

*W_a_, G* and *t* are the the work of adhesion, the shear modulus and the coating thickness, respectively. W_a_ is related to the surface energy (γ), which can be written as:

*W_a_=2γ*

And *G* is related to the elastic modulus (*E*), which can be written as:

*G≈E/3*

Thus, the lower surface energy and elastic modulus of the coating will result in lower ice adhesion strength. The elastic modulus and surface energy of the anti-icing coatings were shown in Fig. S41. The results showed that all the silicone-based coatings illustrated low elastic modulus (< 0.3 MPa) and surface energy (< 20 mJ·m^-2^).

**References**

[1] H. Guo, Y. Han, W. Zhao et al., “Universally autonomous self-healing elastomer with high stretchability,” Nat Commun, vol. 11, pp. 2037, 2020.

[2] W. E. Pickett, “Pseudopotential methods in condensed matter applications,” Computer Physics Reports, vol. 9, pp. 115-197, 1989.

[3] Z. Liu, S. Tian, Q. Li et al., “Integrated dual-functional ormosil coatings with agnps@rgo nanocomposite for corrosion resistance and antifouling applications,” ACS Sustainable Chemistry & Engineering, vol. 8, pp. 6786-6797, 2020.

[4] J. P. Perdew, K. Burke, M. Ernzerhof, “Generalized gradient approximation made simple,” Physical Review Letters, vol. 77, pp. 3865-3868, 1996.

[5] Z. He, W. J. Xie, Z. Liu et al., “Tuning ice nucleation with counterions on polyelectrolyte brush surfaces,” Sci Adv, vol. 2, pp. e1600345, 2016.

[6] R. Lalani, L. Liu, “Synthesis, characterization, and electrospinning of zwitterionic poly(sulfobetaine methacrylate),” Polymer, vol. 52, pp. 5344-5354, 2011.

[7] D. Jiang, Z. Liu, X. He et al., “Polyacrylamide strengthened mixed-charge hydrogels and their applications in resistance to protein adsorption and algae attachment,” RSC Advances, vol. 6, pp. 47349-47356, 2016.

[8] L. An, Y. H. Yu, J. Chen et al., “Synthesis and characterization of tailor-made zwitterionic lignin for resistance to protein adsorption,” Industrial Crops and Products, vol. 167, pp. 113514, 2021.

[9] L.-J. Zhu, L.-P. Zhu, Y.-F. Zhao et al., “Anti-fouling and anti-bacterial polyethersulfone membranes quaternized from the additive of poly(2-dimethylamino ethyl methacrylate) grafted sio2 nanoparticles,” Journal of Materials Chemistry A, vol. 2, pp. 15566-15574, 2014.

[10] T. Shu, J. Daoyi, P. Jibin et al., “A new hybrid silicone-based antifouling coating with nanocomposite hydrogel for durable antifouling properties,” Chemical Engineering Journal, vol. pp. 2019.

[11] L. Guangming, T. Shu, L. Jingyu et al., “Fabrication of bio-based amphiphilic hydrogel coating with excellent antifouling and mechanical properties,” Chemical Engineering Journal, vol. pp. 2020.

[12] A. J. Meuler, J. D. Smith, K. K. Varanasi et al., “Relationships between water wettability and ice adhesion,” ACS Applied Materials & Interfaces, vol. 2, pp. 3100-3110, 2010.
